# Effects of genotype and dietary fish oil replacement with vegetable oil on the intestinal transcriptome and proteome of Atlantic salmon (*Salmo salar*)

**DOI:** 10.1186/1471-2164-13-448

**Published:** 2012-09-04

**Authors:** Sofia Morais, Tomé Silva, Odete Cordeiro, Pedro Rodrigues, Derrick R Guy, James E Bron, John B Taggart, J Gordon Bell, Douglas R Tocher

**Affiliations:** 1 Institute of Aquaculture, University of Stirling, Stirling, FK9 4LA, Scotland, UK; 2 Aquaculture Research Group, Centre of Marine Sciences (CCMAR), University of Algarve, 8005-139, Faro, Portugal; 3 Landcatch Natural Selection Ltd., Cooperage Way Business Centre, Alloa, FK10 3LP, Scotland, UK

## Abstract

**Background:**

Expansion of aquaculture requires alternative feeds and breeding strategies to reduce dependency on fish oil (FO) and better utilization of dietary vegetable oil (VO). Despite the central role of intestine in maintaining body homeostasis and health, its molecular response to replacement of dietary FO by VO has been little investigated. This study employed transcriptomic and proteomic analyses to study effects of dietary VO in two family groups of Atlantic salmon selected for flesh lipid content, 'Lean' or 'Fat'.

**Results:**

Metabolism, particularly of lipid and energy, was the functional category most affected by diet. Important effects were also measured in ribosomal proteins and signalling. The long-chain polyunsaturated fatty acid (LC-PUFA) biosynthesis pathway, assessed by fatty acid composition and gene expression, was influenced by genotype. Intestinal tissue contents of docosahexaenoic acid were equivalent in Lean salmon fed either a FO or VO diet and expression of LC-PUFA biosynthesis genes was up-regulated in VO-fed fish in Fat salmon. Dietary VO increased lipogenesis in Lean fish, assessed by expression of FAS, while no effect was observed on β-oxidation although transcripts of the mitochondrial respiratory chain were down-regulated, suggesting less active energetic metabolism in fish fed VO. In contrast, dietary VO up-regulated genes and proteins involved in detoxification, antioxidant defence and apoptosis, which could be associated with higher levels of polycyclic aromatic hydrocarbons in this diet. Regarding genotype, the following pathways were identified as being differentially affected: proteasomal proteolysis, response to oxidative and cellular stress (xenobiotic and oxidant metabolism and heat shock proteins), apoptosis and structural proteins particularly associated with tissue contractile properties. Genotype effects were accentuated by dietary VO.

**Conclusions:**

Intestinal metabolism was affected by diet and genotype. Lean fish may have higher responsiveness to low dietary n-3 LC-PUFA, up-regulating the biosynthetic pathway when fed dietary VO. As global aquaculture searches for alternative oils for feeds, this study alerts to the potential of VO introducing contaminants and demonstrates the detoxifying role of intestine. Finally, data indicate genotype-specific responses in the intestinal transcriptome and proteome to dietary VO, including possibly structural properties of the intestinal layer and defence against cellular stress, with Lean fish being more susceptible to diet-induced oxidative stress.

## Background

Fish are important components of the human diet, being highly nutritious and valued as the main source of n-3 long-chain polyunsaturated fatty acids (LC-PUFA). These essential fatty acids, mainly eicosapentaenoic acid (EPA) and docosahexaenoic acid (DHA), have well-known health-promoting properties, including protection against a range of cardiovascular and inflammatory diseases, and neurological disorders [1]. With population growth and increasing awareness of the importance of fish consumption as part of a healthy diet, worldwide demand for seafood continues to grow. However, as traditional fisheries are largely in decline, aquaculture must meet this demand. Aquaculture is the fastest-growing food production sector with an average annual growth rate of 6.6%, accounting for 46% of total fish supply [2]. In the European and American continents, aquaculture production is largely dominated by salmonid species, mainly Atlantic salmon, and feeds for such carnivorous species have traditionally relied on fishmeal (FM) and fish oil (FO) from wild stocks. Recent estimates indicated that 88.5% of global production of FO was used by the aquaculture sector, with salmonid culture taking the largest share (56%) [3]. With ever increasing demands for aquafeeds and reduction in fisheries landings, the availability of FO and FM seriously limits the growth of aquaculture production and there is an urgent need to find more sustainable alternatives.

Vegetable oils (VO) can replace FO in salmon feeds without compromising fish growth or condition although, at high levels of replacement, tissue levels of n-3 LC-PUFA are significantly reduced [4,5]. The effects of FO replacement by VO are becoming well characterized in the hepatic transcriptome of salmonids [6-10], and other species [11]. However, studies on intestinal transcriptome are few and restricted to effects of replacement of FM by plant proteins, particularly soybean meal [12,13], given its potential to cause enteritis. It is now clear that intestine in salmonids is not simply a site for reacylation and packaging of dietary lipids but it also has important roles in fatty acid metabolism, including LC-PUFA biosynthesis [14,15]. Furthermore, dietary VO can induce major histological changes in fish enterocytes, originating mostly from supranuclear lipid droplet formation, possibly due to altered reacylation mechanisms and decreased phospholipid synthesis [16-18]. In some cases, these accumulations were large enough to be deemed pathological [19,20]. A recent study investigating effects of dietary FO replacement by VO on intestinal transcriptome in Atlantic cod indicated potential effects on lipid absorption and transport and suggested morphological and structural changes to the intestinal muscle layer [21]. Furthermore, both this and a previous study on Atlantic salmon showed significant effects on expression of genes involved in cell proliferation and apoptosis [22]. Therefore, there is indication that intestine may be affected by changes in lipid components of feed formulations. Given its crucial roles in nutrient absorption, protection against the entry of pathogens, and immune function [23], further attention is warranted and impacts of FO replacement require investigation in intestine, particularly in salmon where important changes in diet formulation are already being applied. This study is a large-scale analysis of the effects of replacement of dietary FO by VO on the transcriptome and proteome of Atlantic salmon intestine. Furthermore, given recent interest in evaluating genetic selection as a feasible strategy, in conjunction with changes in commercial feed formulation, to meet worldwide demand for farmed fish without compromising animal welfare or nutritional value [9,10,24], two groups of Atlantic salmon families, Lean and Fat, were studied to examine the potential effects of genetic background. This experiment was performed in parallel with another microarray study looking at effects in the hepatic transcriptome, analysing samples from the same individuals [9,10], enabling a global and comprehensive assessment of the physiological and molecular effects of FO replacement by VO in Atlantic salmon, including potential interactions with genotype.

## Results

### Microarray analysis

Two way-ANOVA of the cDNA array dataset returned 1409, 1626 and 862 significant genes for the factors diet, genotype and diet × genotype interaction, respectively. Detailed analysis was restricted to the top 100 most significant features, which were categorised according to biological function, based on mammalian homolog genes. Metabolism, particularly of lipid and energy, was the functional category most affected by diet accounting for 39-41% of the top 100 annotated genes (Table 1), and showing highest diet × genotype interaction (Additional file 1). Diet also impacted translation (17%; mostly ribosomal proteins) and signalling (17%). In contrast, genotype affected less markedly metabolism (21%), whereas structural proteins (25%; mostly different types of collagen) and proteins involved in the regulation of transcription (21%) predominated (Table 2).

**Table 1:** Intestine transcripts corresponding to the top 100 most significant features exhibiting differential expression between diets

**Accession no**	**Gene**	**VO/FO**		**p- value**
		**Lean**	**Fat**	
***Metabolism (39%)***				
*Lipid metabolism (9%)*				
DW590668	Fatty aldehyde dehydrogenase	- 1.1	- 1.2	0.0003
CK888998	Ethanolamine kinase 1	1.2	1.2	0.0008
CK884623	Epidermis-type arachidonate lipoxygenase 3	1.4	1.1	0.0010
CK885725	Protein containing a beta-ketoacyl synthase domain	1.4	1.6	0.0017
AF478472	Delta-5 fatty acyl desaturase	1.2	2.5	0.0019
*Energy metabolism/generation of precursor metabolites (11%)*				
CK885194	Cytochrome c oxidase subunit 5A	- 1.2	- 1.1	0.0000
bra_snb_03E12	NADH dehydrogenase subunit 1	- 1.3	- 1.2	0.0000
EG649067	Ubiquinol-cytochrome c reductase core protein 1	- 1.2	- 1.1	0.0004
CK884638	Uncoupling protein	- 1.3	−1.3	0.0019
CK890974	Mitochondrial calcium-dependent solute carrier SCaMC-2	- 1.5	1.0	0.0021
EG648806	NADH dehydrogenase (ubiquinone) 1 beta subcomplex 9	- 1.1	- 1.1	0.0022
*Protein and amino acid metabolism (9%)*				
CK877521	Kunitz-type protease inhibitor 2 precursor	1.2	1.1	0.0002
DW590246	Betaine aldehyde dehydrogenase	- 1.4	- 1.0	0.0005
CK885180	Fumarylacetoacetate hydrolase domain-containing protein 2B	- 1.4	- 1.3	0.0007
CK897653	Ubiquitin-conjugating enzyme E2 A	1.2	1.2	0.0012
CK885145	Lysosomal protective protein precursor (Cathepsin A)	1.1	1.1	0.0014
*Carbohydrate metabolism (5%)*				
EG649263	Endosulfine alpha	- 1.3	- 1.0	0.0005
CK885545	Glucose transporter type 8	1.0	1.5	0.0013
*Xenobiotic and oxidant metabolism (5%)*				
CK885392	Catalase	1.6	1.1	0.0000
CO472279	Cytochrome P450 1^a^	1.2	1.4	0.0004
CK898307	Selenoprotein H-like protein	1.3	1.1	0.0013
***Transport/ intracellular trafficking (5%)***				
EG647578	Calcium-activated potassium channel subunit alpha 1	1.3	1.2	0.0005
EG648841	Sodium-coupled neutral amino acid transporter 2	- 1.2	- 1.2	0.0007
EG648303	Mitochondrial import receptor subunit TOM20 homolog	- 1.1	- 1.1	0.0011
***Regulation of transcription (13%)***				
CK898304	C-Maf	1.6	1.6	0.0000
CK890154	Butyrate response factor 2	1.6	1.2	0.0001
EG647831	Proliferation-associated 2G4 b	- 1.3	- 1.2	0.0003
AM041851	Enhancer trap locus homolog 1	- 1.1	- 1.2	0.0004
CK885179	Steroid receptor RNA activator 1	- 1.3	- 1.3	0.0007
CK884728	Butyrate response factor 1	1.1	1.4	0.0011
CK879187	Enhancer of polycomb homolog 1	1.4	1.1	0.0020
***Translation (17%)***				
CK878380	40S ribosomal protein S18	- 1.1	- 1.2	0.0003
DW591873	60S acidic ribosomal protein	- 1.3	- 1.2	0.0006
EG648644	60S ribosomal protein L29	- 1.1	- 1.2	0.0011
DW590580	40S ribosomal protein S5	- 1.2	- 1.2	0.0011
CK880046	40S ribosomal protein S28	- 1.1	- 1.2	0.0017
EG649167	60S ribosomal protein L37a	- 1.1	- 1.2	0.0017
EG648512	60S ribosomal protein L41	- 1.1	- 1.2	0.0017
CK886042	60S ribosomal protein L31	- 1.1	- 1.3	0.0021
CK895350	60S ribosomal protein L36	- 1.1	- 1.1	0.0021
***Signalling/Signal transduction (17%)***				
CK874863	Proto-oncogene serine/threonine-protein kinase	1.1	1.1	0.0002
CK881266	Mitogen-activated protein kinase kinase kinase kinase 1	1.2	1.2	0.0002
CK878590	Serine/threonine protein kinase (Tribbles homolog 2)	- 1.1	- 1.3	0.0004
AJ425351	Tensin-like C1 domain-containing	- 2.0	- 1.2	0.0006
CK885079	Sonic hedgehog-like protein	1.3	1.0	0.0008
CN181161	TGF-beta type II receptor	1.1	1.1	0.0009
EG649257	Serine/threonine-protein kinase VRK3	- 1.1	- 1.2	0.0009
CK886267	Guanylate cyclase 2C	1.1	1.1	0.0011
CK884467	Serine/threonine-protein phosphatase 2A regulatory subunit B	- 1.4	- 1.3	0.0020
***Structural proteins (7%)***				
mus_snm_13F09	Myosin heavy chain	- 1.2	- 1.1	0.0002
CK885118	Cardiac tropomyosin	1.0	1.1	0.0013
CK883354	Troponin C	1.2	1.2	0.0017
EG647866	Tubulin alpha chain	- 1.2	- 1.2	0.0020
***Immune response (2%)***				
BM414177	Liver-expressed antimicrobial peptide 2	1.5	1.4	0.0022
***Miscellaneous***				
CK884634	Ezrin-radixin-moesin binding phosphoprotein 50	1.1	1.1	0.0003
EG648821	Transposase	- 1.1	- 1.2	0.0004
CK883634	Poly [ADP-ribose] polymerase 12	1.3	1.1	0.0006
CK885090	Claudin 15	1.2	1.1	0.0008
DW589291	Transposase	- 1.3	- 1.3	0.0009
CK878888	Splicing factor, arginine/serine-rich 3	- 1.3	- 1.2	0.0013
CK885098	RNA-binding protein with multiple splicing 2	- 1.4	- 1.2	0.0013
CK891747	Occludin	- 1.2	- 1.2	0.0014
CK888908	All-trans-retinol 13,14-reductase precursor	1.1	1.2	0.0017
CK880622	Cyclin G1	- 1.1	- 1.2	0.0019
CK885604	Methylenetetrahydrofolate dehydrogenase (NADP+ dependent) 1-like	- 1.6	- 1.3	0.0020
CK891699	Barrier-to-autointegration factor	- 1.1	- 1.2	0.0020
CK879123	Hemoglobin subunit beta	1.5	1.0	0.0022

Annotated features (67% of all clones) are arranged by categories of biological function (with percentages of distribution of genes within each category being indicated) and, within these, by decreasing significance (assessed by two-way ANOVA). Also indicated are the GenBank accession numbers for each clone (or, when not available, the probe number) and the expression ratio between fish fed VO and those fed FO, for each genotype (Lean and Fat).

**Table 2:** Intestine transcripts corresponding to the top 100 most significant features exhibiting differential expression between family groups

**Accession no**	**Gene**	**Lean/Fat**		**p- value**
		**FO**	**VO**	
***Metabolism (21%)***				
*Lipid metabolism (5%)*				
CK874747	Acylglycerol kinase	- 1.0	- 1.1	0.0003
BI468033	ATP-binding cassette sub-family A member 1	- 1.1	- 1.6	0.0004
*Energy metabolism/generation of precursor metabolites (2%)*				
CK890974	Mitochondrial calcium-dependent solute carrier SCaMC-2	- 1.1	- 1.6	0.0000
*Protein and amino acid metabolism (12%)*				
EG647485	Proteasome subunit beta type-8 precursor	- 1.3	- 1.8	0.0000
DW591059	Heat shock protein Hsp-16	1.2	1.4	0.0001
AJ425553	26S proteasome non-ATPase regulatory subunit 9	- 1.2	- 1.1	0.0002
DW592187	26 proteasome complex subunit DSS1	1.5	1.3	0.0008
CK878109	Heat shock protein 60 kDa	1.2	1.4	0.0009
*Xenobiotic and oxidant metabolism (2%)*				
CO472279	Cytochrome P450 1A	- 1.2	- 1.5	0.0003
***Transport/ intracellular trafficking (5%)***				
CK886307	Peptide transporter PEPT2	- 1.4	- 1.3	0.0006
DW588251	Post-GPI attachment to proteins factor 2	1.1	1.3	0.0007
***Regulation of transcription (21%)***				
CK877881	Forkhead box Q1	- 1.4	- 1.7	0.0000
CK884548	Proline-rich nuclear receptor coactivator 2	- 1.1	- 1.2	0.0001
CK885013	Forkhead box protein F1	1.1	1.5	0.0003
CK891975	Y-box binding protein-2	- 1.1	- 1.1	0.0003
DW589341	Signal transducer/activator of transcription Stat1	- 1.2	- 1.2	0.0004
CN181282	Bromodomain containing protein 3	- 1.1	- 1.1	0.0007
CK884407	C-Myc-binding protein	- 1.3	- 1.1	0.0007
CK895950	Transcription factor CP2-like	- 1.5	- 1.2	0.0007
EG648473	BTB/POZ domain-containing protein	- 1.1	- 1.4	0.0009
***Translation (7%)***				
CN181322	40S ribosomal protein S4	1.1	1.1	0.0002
CK885979	Ribonuclease UK114	- 1.1	- 1.3	0.0005
EG648042	Eukaryotic translation initiation factor 3, subunit J	1.1	1.1	0.0009
***Signalling/Signal transduction (16%)***				
CO471793	Rho GDP-dissociation inhibitor 1	1.3	1.0	0.0000
CK885560	Protein tyrosine phosphatase non-receptor type 9	1.3	1.5	0.0004
CK885079	Sonic hedgehog-like protein	- 1.3	- 1.0	0.0004
EG648038	Kalirin, RhoGEF kinase	1.2	1.2	0.0005
DW589610	Chimaerin 1	1.2	1.2	0.0005
CK897590	Growth factor receptor-bound protein 2	1.1	1.1	0.0009
EG647545	Protein tyrosine phosphatase receptor type D	1.0	1.2	0.0009
***Structural proteins (25%)***				
CK882401	Troponin C	- 1.1	- 1.2	0.0000
CK889952	Transgelin 2	1.5	1.7	0.0000
EG648135	Osteonectin	1.3	1.5	0.0000
CK899058	Collagen alpha 3 type VI	1.5	1.5	0.0002
CK879405	Collagen alpha 2 type VI	1.3	1.5	0.0002
EG649361	Collagen alpha 2 type I	1.5	1.7	0.0005
EG649013	Collagen alpha 3 type I	1.4	1.5	0.0005
mus_mfo_15A10	Troponin-I isoform 3	1.4	1.6	0.0007
CK876833	Keratocan	- 1.0	1.6	0.0007
DW590534	Collagen alpha 2 type I	1.5	1.6	0.0010
CK892271	Collagen alpha 2 type V	1.2	1.4	0.00010
CK873441	Collagen alpha1 type VI	1.2	1.4	0.0010
***Immune response (5%)***				
AJ425527	T-cell immunoglobulin and mucin domain-containing protein 4	1.1	1.1	0.0001
EG649194	Mannose-binding protein C	1.4	1.4	0.0003
***Miscellaneous***				
AM041770	Preimplantation protein 3	- 1.3	- 1.2	0.0000
EG649106	Ornithine decarboxylase antizyme 2	1.4	1.1	0.0002
CK884355	Guanylin precursor	1.2	1.4	0.0002
CK879185	Aminolevulinate, delta-, synthetase 1	- 2.0	- 1.1	0.0002
CK885814	Similar to p53-associated parkin-like cytoplasmic protein	1.2	1.1	0.0002
CK886214	Septin-7	1.1	1.1	0.0003
BM414079	Tumor protein D52-like 2	- 1.1	- 1.3	0.0003
BM413818	Splicing factor, proline- and glutamine-rich	1.2	1.2	0.0003
CK874550	Follistatin-related protein 1	1.1	1.3	0.0004
DW589104	Nucleoside diphosphate kinase 6	1.1	1.1	0.0005
DW592207	Deoxyhypusine synthase	1.5	1.6	0.0007
CK892359	HCLS1-associated protein X-1	- 1.2	- 1.1	0.0007
BI468158	Nuclear protein 1	- 1.0	- 1.6	0.0009
AJ425003	Lamina-associated polypeptide 2	1.2	1.1	0.0010
CK899283	Small EDRK-rich factor 2	1.3	1.5	0.0010
CK885090	Claudin 15	- 1.2	- 1.1	0.0011

Annotated features (60% of all clones) are arranged by categories of biological function (with percentages of distribution of genes within each category being indicated) and, within these, by decreasing significance (assessed by two-way ANOVA). Also indicated are the GenBank accession numbers for each clone (or, when not available, the probe number) and the expression ratio between Lean and Fat fish fed either FO or VO.

Gene Ontology (GO) enrichment analysis was performed on the complete significant lists, enabling identification of GO terms significantly enriched in the input entity list, in comparison to the whole array, providing clues as to which biological processes might be particularly altered in the experimental conditions being compared. It revealed no significant enrichment of GO terms in the genotype list, while 20 and 7 GO terms were significantly enriched in the diet and interaction lists, respectively. GO terms enriched in the diet list included structural constituents of ribosome, structural molecule activity, cytosolic ribosome, cytosol, ribosomal subunit, translation, cellular biosynthetic process, gene expression, macromolecule and biopolymer biosynthetic process and other related terms. This was explained by the large number of ribosomal proteins, components of both the 40S and 60S subunits, which were down-regulated by dietary VO (Table 1). In contrast, several Δ6-desaturase (Δ6fad) clones showing a diet × genotype interaction (see Additional file 1) caused a significant enrichment of the GO terms oxidoreductase activity, stearoyl-CoA 9-desaturase activity, unsaturated fatty acid biosynthetic activity/metabolic processes and very long chain fatty acid (VLCFA) biosynthetic activity/metabolic processes.

### RT-qPCR analysis of gene expression

The expression of several genes significantly affected or related to processes affected by the two factors in the microarray analysis was determined by RT-qPCR (Table 3). For diet, a reasonably good match was found for Δ5 fatty acyl desaturase (Δ5fad), NADH dehydrogenase subunit 1 (NADH1), proliferation-associated 2G4b (PA2G4), 60S acidic ribosomal protein (RP60S), proliferating cell nuclear antigen (PCNA; also known as cyclin) and cytochrome P450 1A (CYP1A), particularly in the Fat group where fold-changes were generally more pronounced and significant. No change in expression of uncoupling protein 2 (UCP2) with diet was measured while, for myosin heavy chain (MYO) and methylenetetrahydrofolate dehydrogenase (NADP+ dependent) 1-like (MTHFD1), RT-qPCR indicated a change opposite to that suggested by microarray. Regarding genotype, a good match was obtained for CYP1A, proteasome subunit beta type-8 precursor (PSMB8) and alpha 2 type I collagen (COL1A2), while transgelin 2 (TAGLN) expression did not differ between family groups, and for ATP-binding cassette sub-family A member 1 (ABCA1) there was an inverse change in expression.

**Table 3:** Relative expression of genes assayed by RT-qPCR

**Genes**	**VO/FO**				**Lean/Fat**			
	**Lean**		**Fat**		**FO**		**VO**	
	**Ratio**	**p value**	**Ratio**	**p value**	**Ratio**	**p value**	**Ratio**	**p value**
**Microarray validation**								
Δ5fad (D)	1.72	ns	**4.34**	**0.005**	2.49	ns	−1.01	ns
NADH1 (D)	−1.03	ns	**−1.28**	**0.05**	1.00	ns	1.24	ns
UCP2 (D)	−1.10	ns	1.08	ns	−1.10	ns	**−1.28**	**0.003**
MTHFD1 (D)	1.20	ns	−1.09	ns	−1.08	ns	1.22	ns
PA2G4 (D)	−1.27	ns	**−1.23**	**0.032**	1.11	ns	1.1	ns
RP60S (D)	−1.02	ns	**−1.69**	**0.023**	1.13	ns	**1.9**	**0.006**
MYO (D)	**1.77**	**0.034**	1.22	ns	−1.35	ns	1.08	ns
PCNA (D)	−1.14	ns	−1.27	ns	1.08	ns	1.20	ns
CYP1A (D+G)	**1.71**	**0.004**	**1.81**	**0.023**	**−2.22**	**0.003**	**−2.38**	**0.001**
ABCA1 (G)	1.24	ns	1.17	ns	1.21	ns	**1.28**	**0.015**
PSMB8 (G)	−100.00	ns	**1.87**	**0.033**	**−16.67**	**0.026**	**< −1000**	**0.000**
TAGLN (G)	1.23	ns	−1.02	ns	−1.03	ns	1.22	ns
COL1A2 (G)	1.31	ns	−1.06	ns	1.47	ns	**2.06**	**0.001**
**LC-PUFA biosynthesis and regulation**								
Δ6fad_a	1.11	ns	**2.96**	**0.008**	1.76	ns	−1.52	ns
elovl5a	1.08	ns	1.2	ns	1.03	ns	−1.08	ns
elovl5b	−1.19	ns	**1.92**	**0.031**	1.54	ns	−1.47	ns
elovl2	1.2	ns	**14.74**	**0.003**	4.89	ns	−2.50	ns
**Fatty acid synthesis**								
FAS	**1.49**	**0.010**	1.20	ns	1.05	ns	1.31	ns
**Fatty acid oxidation**								
ACO	1.18	ns	1.28	ns	1.19	ns	1.09	ns
CPT1	1.23	ns	−1.09	ns	−1.16	ns	1.15	ns
**Regulation of lipid metabolism**								
PPARα	1.14	ns	1.38	ns	1.26	ns	1.04	ns
PPARβ	1.03	ns	1.03	ns	−1.02	ns	−1.02	ns
PPARγ	1.52	ns	**1.60**	**0.036**	1.21	ns	1.15	ns
**Xenobiotic and oxidative stress**								
GST	−1.30	ns	1.07	ns	1.21	ns	−1.14	ns
MT-A	−1.82	0.051*	−1.05	ns	−1.25	ns	**−2.13**	**0.007**
CAT	**1.57**	**0.036**	1.26	ns	−1.52	ns	−1.22	ns
SOD	−1.20	ns	−1.04	ns	1.21	ns	1.04	ns
**Apoptosis**								
CASP3B	**1.22**	**0.045**	1.22	ns	−1.04	ns	−1.04	ns
CASP6A/B	1.30	ns	1.37	ns	−1.14	ns	−1.20	ns

fad: Fatty acyl desaturase (Δ5 and Δ6 activities); NADH1: NADH dehydrogenase subunit 1; UCP2: Uncoupling protein 2; elovl: MTHFD1: Methylenetetrahydrofolate dehydrogenase (NADP+ dependent) 1-like; PA2G4: Proliferation-associated 2G4b; RP60S: 60S acidic ribosomal protein; MYO: Myosin heavy chain; PCNA: Proliferating cell nuclear antigen; CYP1A: Cytochrome P4501A; ABCA1: ATP-binding cassette sub-family A member 1; PSMB8: Proteasome subunit beta type-8 precursor; TAGLN: Transgelin 2; COL1A2: Collagen type I alpha 2; elovl2: Elongase 2; FAS: Fatty acid synthase; ACO: Acyl-CoA oxidase; CPT1: Carnitine palmitoyltransferase I; PPARs; Peroxisome proliferator-activated receptors; GST: glutathione S-transferase; MT-A: Metallothionein A; CAT: catalase; SOD: superoxide dismutase; CASP; caspase.

*Only marginally non-significant.

Genes correspond to features significantly affected by either diet (D) or genotype (G) in the microarray analysis or involved in biological processes being analysed in further detail. Values are normalized (by *cofilin-2* and *elf-1 α*) gene expression ratios between fish fed VO in relation to FO for each family group or of Lean fish in relation to the Fat group when fed either one of the diets. Values in bold are significantly different, at p<0.05 (REST 2008); ns - non-significant.

As well as validation above, RT-qPCR was used to further analyse genes/pathways identified by microarray and published data as potentially interesting, including lipid metabolism, xenobiotic and oxidative stress, and apoptosis. One was LC-PUFA biosynthesis, given that Δ5fad was significantly affected by diet in the microarray analysis, with a stronger response in Fat fish, whereas Δ6fad showed a significant diet × genotype interaction (supplementary file 1) confirmed by RT-qPCR. The Δ6fad transcript was only significantly up-regulated in Fat fish fed VO, compared to FO, and Lean fish showed higher levels of Δ6fad expression than Fat fish when fed FO, while the opposite trend was noted when fish were fed VO. Fatty acyl elongases (elovl’s) were also quantified and their expression (except elovl5a) broadly followed that of fads: significantly up-regulated when dietary VO replaced FO in the Fat group. Additionally, elovl5b and, particularly, elovl2 showed a trend for increased expression (high fold-change) in Lean fish, compared to Fat fish, when fed FO, while an opposite trend was observed when salmon were fed VO. Although genes involved in fatty acid synthesis and oxidation showed few significant differences, expression of fatty acid synthase (FAS) was up-regulated in fish fed VO, but only in Lean fish. The expression of peroxisome proliferator-activated receptors (PPARs), involved in the regulation of multiple lipid metabolism genes, was determined but only PPARγ showed any significant change, being up-regulated in the Fat group when dietary VO replaced FO. Of the xenobiotic and oxidative metabolism genes assayed, apart from CYP1A, only catalase (CAT) was affected by diet (up-regulated in fish fed VO) and only significantly in the Lean family. In contrast, metallothionein A (MT-A) showed higher expression in Fat fish, but only when fed VO, while a marginal down-regulation was observed when comparing VO and FO-fed fish in the Lean group. Of genes related to apoptosis, CASP3B was up-regulated by VO in Lean fish whereas a similar fold-change was marginally non-significant in the Fat fish.

### Intestine fatty acid composition

The levels of most fatty acids in pyloric caeca were affected by diet, whereas genotype had no significant effect (Table 4). However, some fatty acids also showed a significant diet × genotype interaction, indicating that the effect of diet depended on the genetic background of the fish. For instance, interactions were observed for some LC-PUFA as a result of higher levels being found in the Lean group, compared to Fat, when fish were fed VO, while the reverse was observed when fed FO. Another unexpected result was that similar levels of DHA in FO- and VO-fed Lean fish meant that, in spite of substantial differences in Fat fish fed the two diets, the effect of diet on DHA was marginally non-significant. Similarly, levels of EPA and 22:5n-3 between FO- and VO-fed fish were noticeably closer in the Lean group.

**Table 4:** Fatty acid compositions (% of total fatty acids) and lipid contents (% of wet weight) of intestinal tissue of Lean and Fat family groups fed diets containing either FO or VO

	**FO**		**VO**		**Two-way ANOVA**		
	**Fat**	**Lean**	**Fat**	**Lean**	**D**	**G**	**D×G**
Fatty acid							
Total saturated	23.70 ± 1.21	22.82 ± 1.09	18.57 ± 0.73	20.08 ± 1.38	0.000	0.742	0.066
Total monoenes	38.25 ± 7.15	44.45 ± 5.31	44.98 ± 2.38	39.34 ± 4.49	0.717	0.928	0.046
18:2n-6	4.19 ± 0.67	4.51 ± 0.24	13.14 ± 0.59	11.28 ± 1.12	0.000	0.139	0.041
18:3n-6	0.10 ± 0.02	0.10 ± 0.02	0.28 ± 0.12	0.25 ± 0.13	0.003	0.611	0.611
20:2n-6	0.57 ± 0.06	0.58 ± 0.07	1.54 ± 0.40	1.65 ± 0.06	0.000	0.199	0.510
20:3n-6	0.17 ± 0.06	0.14 ± 0.02	1.01 ± 0.12	1.47 ± 0.26	0.000	0.056	0.019
20:4n-6	1.20 ± 0.60	0.85 ± 0.31	0.74 ± 0.18	1.40 ± 0.29	0.785	0.419	0.016
22:4n-6	0.08 ± 0.06	0.05 ± 0.06	0.00 ± 0.00	0.09 ± 0.07	0.337	0.337	0.038
22:5n-6	0.21 ± 0.06	0.16 ± 0.07	0.07 ± 0.02	0.12 ± 0.05	0.002	1.000	0.128
Total n-6 PUFA	6.51 ± 0.18	6.38 ± 0.36	16.79 ± 0.86	16.27 ± 0.67	0.000	0.410	0.781
18:3n-3	1.46 ± 0.38	1.67 ± 0.21	4.79 ± 0.53	3.71 ± 0.75	0.000	0.211	0.021
18:4n-3	1.36 ± 0.60	1.67 ± 0.66	0.85 ± 0.31	0.55 ± 0.20	0.002	0.712	0.127
20:3n-3	0.27 ± 0.05	0.33 ± 0.14	0.67 ± 0.18	0.62 ± 0.04	0.000	0.768	0.384
20:4n-3	1.77 ± 0.28	2.16 ± 0.46	0.97 ± 0.13	0.83 ± 0.10	0.000	0.504	0.061
20:5n-3	6.55 ± 0.55	5.79 ± 0.62	3.60 ± 0.90	4.40 ± 0.72	0.000	0.682	0.058
22:5n-3	2.82 ± 0.81	2.30 ± 0.74	1.57 ± 0.36	2.54 ± 0.77	0.157	0.530	0.052
22:6n-3	16.68 ± 6.68	11.65 ± 5.65	6.97 ± 1.58	11.50 ± 3.10	0.057	0.943	0.050
Total n-3 PUFA	30.90 ± 5.94	25.56 ± 5.19	19.41 ± 2.18	24.15 ± 3.76	0.015	0.962	0.042
Total PUFA	38.06 ± 5.96	32.73 ± 5.42	36.45 ± 2.08	40.59 ± 3.16	0.206	0.794	0.059
Total lipids	7.50 ± 3.75	9.56 ± 3.81	6.97 ± 1.07	4.59 ± 1.05	0.081	0.804	0.111

Results are means ± SD (n = 4) and p-values of two-way ANOVA are presented for diet (D), genotype (G) and interaction between both factors.

### Proteomic analysis

**Figure 1:**
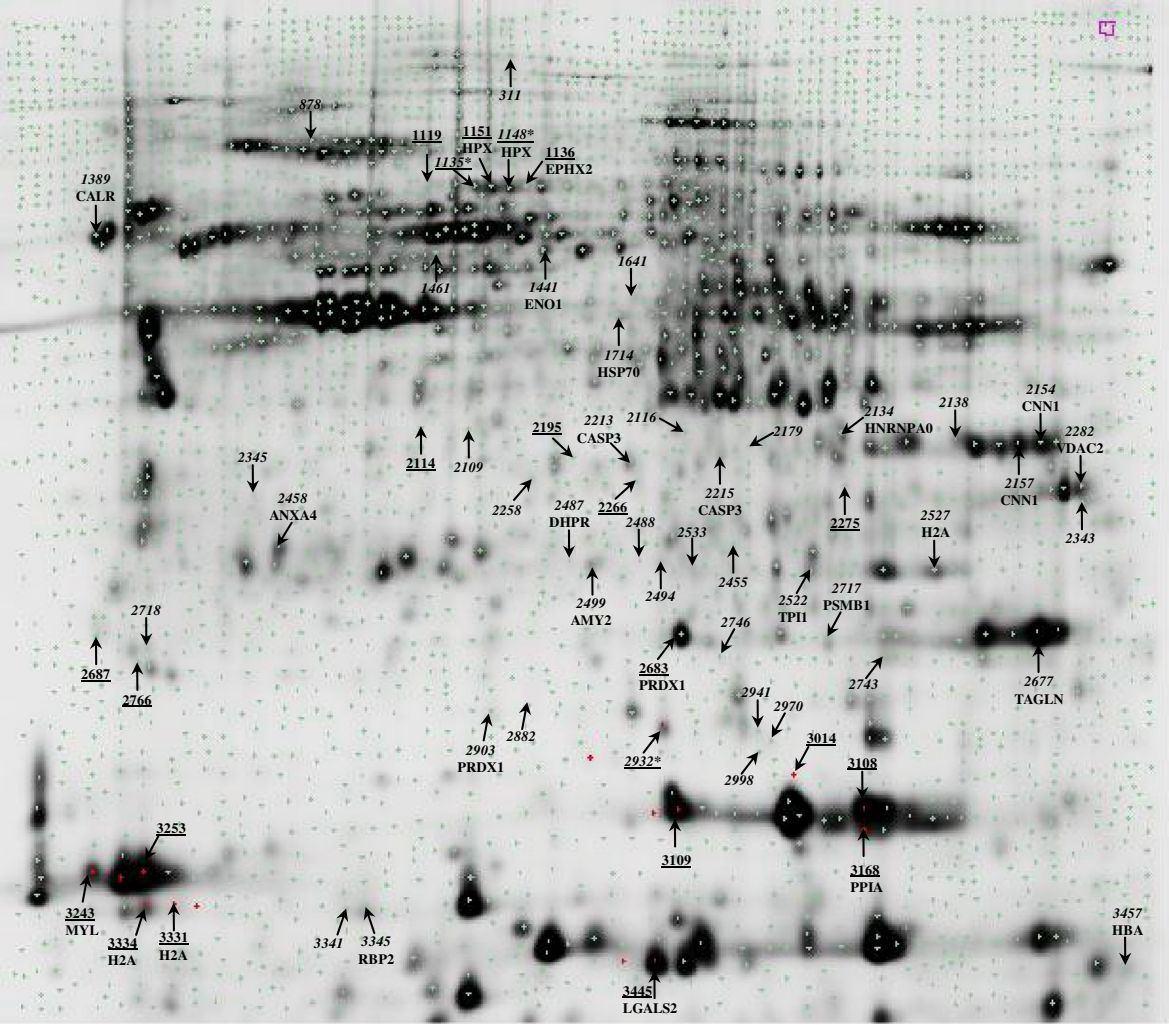
**Proteins significantly affected by diet or genotype.** Cropped image of a scanned 2-D polyacrylamide gel showing all spots identified as being differentially expressed (P<0.05, two-way ANOVA) in fish fed either FO or VO (underlined spot numbers), or between family groups (remaining spot numbers); the three underlined spot numbers containing an * were differentially affected by both diet and genotype. Spots that returned reliable identifications by peptide fragment fingerprinting are identified by their abbreviation

Of the protein spots identified as being differentially expressed between diets or genotypes (Additional files 2 and 3), only 17 and 29 could be excised and, of these, only 9 and 20, respectively, returned reliable identifications by peptide fragment fingerprinting (Figure 1). Proteins significantly up-regulated by dietary VO are likely implicated in xenobiotic/drug metabolism (epoxide hydrolase 2, EPHX2), protection from oxidative stress (hemopexin-like protein, HPX and peroxiredoxin-1, PRDX1) and induction of apoptosis and inflammatory responses (galectin-2, LGALS2). Those proteins down-regulated by dietary VO included proteins responsible for protein folding and involved in signalling (2-peptidylprolyl isomerase A, PPIA), actin-based motility (myosin light chain, MYL) and DNA replication, repair or transcription (histone H2A, H2A) (Table 5). Proteins affected by genotype encompass a variety of pathways, of which only a few are related to metabolism, namely carbohydrate (alpha-enolase, ENO1; pancreatic alpha-amylase precursor, AMY2; triosephosphate isomerase 1b, TPI1), folate (dihydropteridine reductase, DHPR) or retinol (retinol-binding protein II, RBP2) metabolism (Table 6). Other proteins may have potentially multiple roles but can broadly be assigned roles in response to oxidative and cellular stress (HPX; PRDX; heat shock protein 70, HSP70), oxygen transport (alpha globin, HBA), signal transduction (calreticulin, CALR), transcription/RNA repair (heterogeneous nuclear ribonucleoprotein A0, HNRNPA0; histone H2A, H2A), apoptosis (CASP3), cellular transport, potentially also associated with apoptosis (voltage-dependent anion channel 2–2, VDAC2; annexin A4, ANX4), and proteolysis (proteasome beta 1 subunit, PSMB1). As with the microarray analysis, a few proteins with a more structural function and particularly associated with tissue contractile properties were affected by genotype, showing lower levels in Lean fish. These included calponin-1 (CNN1) and transgelin (TAGLN), the latter which was also found to be significantly affected by microarray, albeit up-regulated in Lean fish. Most proteins significantly affected by genotype showed lower levels of expression in the Lean group, with the exception of ENO1, HSP70, TPI1, H2A and HBA.

**Table 5:** Proteins differentially regulated by diet

**Spot No**	**Protein ID**	**Accession No. (NCBInr)**	**Theoretical Mw (KDa)/pI**	**Protein Score**^**a**^	**No. Matched peptides (MS/MS)**^**b**^	**Best score peptide**	**VO/FO Lean**	**VO/FO Fat**	**p-value**
1136	Epoxide hydrolase 2 (EPHX2)	gb|ACI33129.1|	54.2/5.52	84	1	GGLFVGLPDEIPR	1.23	1.40	0.0190
1148	Hemopexin-like protein* (HPX)	emb|CAA92147.1|	50.4/5.61	169	1	VHLDAITSDDAGNIYAFR	1.32	1.56	0.0002
1151	Hemopexin-like protein* (HPX)	ref|NP_001104617.1|	51.0/6.18	161	1	VHLDAITSDDAGNIYAFR	1.38	1.54	0.0001
2683	Peroxiredoxin 1 (PRDX1)	gb|ACI67145.1|	22.0/6.42	757	8	SISTDYGVLKEDEGIAYR	1.14	1.09	0.0310
3168	2-peptidylprolyl isomerase A (PPIA)	ref|NP_001135161.1|	17.5/7.61	101	1	VYFDITIGDTPAGR	−1.46	−1.18	0.0170
3243	Myosin light chain smooth muscle isoform (MYL)	ref|NP_998803.1|	16.9/4.47	100	2	EAFLLFDR	−1.23	−1.11	0.0490
3331	similar to H2A histone family, member J (H2A)	ref|XP_001521566.1|	13.3/10.84	74	1	AGLQFPVGR	−1.58	−1.18	0.0250
3334	Histone H2A** (H2A)	emb|CAA25528.1|	13.7/10.88	37	1	AGLQFPVGR	−1.48	−1.18	0.0210
3445	Galectin 2 (LGALS2)	gb|ACN10131.1|	14.8/5.93	314	2	SGASSFSINIGHDSDNYALHFNPR	1.17	1.15	0.0170

^a^ The protein score probability limit (where P < 0.05) is 73.

^b^ Peptides with confidence interval above 95% were considered.

* Equivalent to warm-temperature-acclimation-related-65 kDa-protein.

**Same protein identification obtained by MS/PMF (peptide mass fingerprinting): Score = 76; 7 peptides matched.

Only reliable identifications of Actinopterygii, obtained by mass spectrometry (MALDI-TOF-MS/MS) analysis and searches in MASCOT, NCBI and ExPASy (Mw/pI) databases are shown. Expression ratios between fish fed VO and FO diets are given for each family group, as well as p-value for diet (two-way ANOVA, DeCyder V7.0).

**Table 6:** Proteins differentially regulated by genotype

**Spot No**	**Protein ID**	**Accession No. (NCBInr)**	**Theoretical Mw (KDa)/pI**	**Protein Score**^**a**^	**No. Matched peptides (MS/MS)**^**b**^	**Best score peptide**	**Lean/Fat FO**	**Lean/Fat VO**	**p-value**
1148	Hemopexin-like protein* (HPX)	emb|CAA92147.1|	50.4/5.61	169	1	VHLDAITSDDAGNIYAFR	−1.09	−1.29	0.0270
1389	Calreticulin precursor (CALR)	gb|ACI32936.1|	47.6/4.33	81	2	FEPFSNEGK	−1.28	−1.33	0.0026
1441	Alpha-enolase (ENO1)	ref|NP_001133366.1|	47.0/5.91	405	3	AAVPSGASTGIYEALELR	1.28	1.2	0.0066
1714	Heat shock protein 70 (HSP70)	ref|NP_990334.1|	70.8/5.47	111	1	IINEPTAAAIAYGLDKK	1.16	1.77	0.0260
2134	Heterogeneous nuclear ribonucleoprotein A0 (HNRNPA0)	gb|ACI67551.1|	28.8/9.10	235	2	LFVGGLNVDTDDDGLRK	−1.19	−1.09	0.0460
2154	Calponin 1 (CNN1)	ref|NP_001139857.1|	33.2/8.56	565	4	KINTSPQNWHQLENIGNFVR	−1.34	−1.51	0.0350
2157	Calponin 1*** (CNN1)	ref|NP_001139857.1|	33.2/8.56	43	1	YDPQKEEELR	−1.34	−1.47	0.0240
2213	Caspase 3 (CASP3)	ref|NP_001133393.1|	31.0/5.97	254	2	IPVEADFLYAYSTAPGYYSWR	−1.15	−1.24	0.0300
2215	Caspase 3 (CASP3)	ref|NP_001133393.1|	31.0/5.97	279	2	VANDQTVQQIQQLLSK	−1.14	−1.31	0.0130
2282	Voltage-dependent anion channel 2–2 (VDAC2)	gb|ACH71030.1|	30.1/8.85	214	1	VNNNSLVGVGYTQTLRPGVK	−1.24	−1.19	0.0160
2458	Annexin A4 (ANXA4)	gb|ACI69495.1|	28.4/5.22	218	3	NHLLQVFK	−1.14	−1.11	0.0300
2487	Dihydropteridine reductase (DHPR)	gb|ACI67281.1|	15.7/8.46	81	1	QSVWTSTISSHLATR	−1.14	−1.11	0.0300
2499	Pancreatic alpha-amylase precursor (AMY2)	ref|NP_001036176.1|	57.4/6.89	86	1	ALVFVDNHDNQR	−1.37	−1.27	0.0021
2522	Triosephosphate isomerase 1b (TPI1)	ref|NP_001133174.1|	26.6/7.63	336	2	LDPNTEVVCGAPSIYLEFAR	1.26	1.16	0.0009
2527	Histone cluster 1 (H2A)	ref|NP_001086775.1|	14.0/10.88	94	1	AGLQFPVGR	1.12	1.11	0.0074
2677	Transgelin (TAGLN)	gb|ACM09025.1|	21.7/7.69	219	1	DGCVLSELINSLHK	−1.61	−1.35	0.0350
2717	Proteasome beta 1 subunit (PSMB1)	ref|NP_001003889.1|	26.1/6.32	87	1	GAVYSFDPVGSYQR	−1.29	−1.12	0.0140
2903	Peroxiredoxin-1(PRDX1)	gb|ACI67145.1|	22.0/6.42	115	1	QITINDLPVGR	−1.62	−1.99	0.0069
3345	Retinol-binding protein II, cellular (RBP2)	ref|NP_001139954.1|	15.6/5.44	70	2	AIDIDFATR	−1.32	−1.1	0.0330
3457	Alpha globin (HBA)	emb|CAA65949.1|	15.1/9.19	170	2	TYFSHWADLSPGSAPVK	1.03	2.09	0.0320

^a^The protein score probability limit (where P < 0.05) is 73.

^b^ Peptides with confidence interval above 95% were considered.

* Equivalent to warm-temperature-acclimation-related-65 kDa-protein.

*** Same protein identification obtained by MS/PMF (peptide mass fingerprinting): Score = 193; 27 peptides matched.

Only reliable identifications of Actinopterygii, obtained by mass spectrometry (MALDI-TOF-MS/MS) analysis and searches in MASCOT, NCBI and ExPASy (Mw/pI) databases are shown. Expression ratios between Lean and Fat family groups are given for each dietary treatment, as well as p-value for genotype (two-way ANOVA, DeCyder V7.0).

## Discussion

Dietary plant ingredients can induce chronic intestinal inflammatory conditions in salmonids that can ultimately result in carcinogenesis [25]. This extreme reaction is rare and usually associated with soy protein at high levels [26]. Dietary n-3 LC-PUFA have important anti-inflammatory and anti-carcinogenic effects in mammalian intestine [23]. Therefore, use of feeds containing high percentages of plant proteins combined with replacement of FO by VO, as is now prevalent in the industry, requires studies on dietary effects on intestinal transcriptomes and proteomes. However, interpretation of the data was difficult as the effects on dietary treatments and/or family groups were subtle, as also observed in liver transcriptome [9], and is typical of this type of experiment [6,8,11,27]. Partly as a consequence, validation of the microarray data gave variable results, from perfect match, to opposite changes in a few, although effects observed in the microarray, with fold changes as low as 1.2 (e.g., NADH1 and PA2G4) were validated by RT-qPCR. In view of the whole genome duplication event that occurred in salmonids [28], gene expression studies are often more challenging due to the presence of highly similar genes which may hybridize with cDNA probes presenting low specificity, further complicated if similar transcripts, corresponding to duplicated genes, are differentially regulated [10]. Nonetheless, the presence of several features related to specific processes in both the transcriptomic and proteomic analysis gave supporting evidence to the pathways likely differentially affected by dietary oil and genetic background related to flesh adiposity.

### Intestinal LC-PUFA biosynthesis capacity is differentially affected by diet and genotype

Considering whether genetic selection for fish families showing better adaptation to more sustainable feeds might be a viable approach to develop aquaculture, one outcome of this investigation was to establish if effects of diet on expression of LC-PUFA biosynthesis genes depended on genotype, as shown in the liver transcriptome of these fish [9]. This was not seen in the hepatic transcriptome of European sea bass families showing different growth rates when fed a vegetable diet but, in this, case similar LC-PUFA profiles were also noted in both genotypes in response to the vegetable diet [11]. In both salmon tissues, differences in n-3 LC-PUFA content between fish fed FO or VO were smaller in the Lean family group. This was due to higher levels of n-3 LC-PUFA in Lean salmon, compared to Fat, when fish were fed VO, but higher amounts in the Fat family group when fed FO. However in liver, up-regulation of LC-PUFA biosynthesis when fish were fed VO was much larger in the Lean family group, whereas in intestine the same individuals only showed significant up-regulation in Fat fish. This appears contradictory but can be explained by the differential tissue n-3 LC-PUFA contents. Although the difference was smaller in Lean fish compared to Fat fish in both tissues, in liver there was still a considerable difference in n-3 LC-PUFA levels between fish fed FO or VO, while in intestine levels were similar. PUFA have important activities on transcription factors, either as direct ligands or through effects on membrane composition [29], affecting transcription of many genes involved in lipid metabolism, including desaturases and elongases [30,31]. In salmon, regulation of genes of LC-PUFA biosynthesis that are known to respond to dietary composition, i.e., Δ5fad, Δ6fad, elovl5b and elovl2 [32-34], appear to show high plasticity and are likely under feedback regulation by tissue n-3 LC-PUFA. Both studies suggest that the Lean family group may show an enhanced response to low dietary n-3 LC-PUFA, with greater up-regulation of biosynthesis when fed VO. In contrast to liver, this response was sufficient in intestine to maintain tissue n-3 LC-PUFA, particularly DHA, at similar levels to fish fed FO. Considering that differences in desaturase expression between the Fat and Lean fish were only significant when FO but not VO, was fed, suggests that the likely mechanism is through negative feedback by high levels of n-3 LC-PUFA rather than positive feedback from low levels of LC-PUFA and/or higher levels of shorter chain precursors.

### Other dietary effects on lipid metabolism

Transcriptional regulation of desaturases and elongases by LC-PUFA may involve both PPARα and sterol regulatory element binding protein 1c (SREBP-1c) [30,31]. In liver, expression of Δ5fad, Δ6fad, elovl2, PPARα, and possibly PPARβ, appeared co-ordinately regulated by diet depending on genotype, while PPARγ was not affected [9]. In intestine, however, expression of PPARα and PPARβ was not affected by either diet or genotype, while PPARγ was up-regulated by dietary VO, significantly in Fat fish. This suggests that dietary regulation of lipid metabolism genes in fish intestine might differ to mammals, where PPARα showed differential expression in response to dietary EPA and DHA in murine intestine [35]. Reasons for differential regulation of PPARs between salmon liver and intestine are unclear, but may be due to different patterns of tissue expression. In plaice and seabream, there was no nutritional regulation (fasting/feeding) of PPARs in the intestine, where PPARγ was the dominant isotype, in contrast to liver where PPARα was dominant [36]. PPARγ in both mammals and fish is predominantly expressed in adipose tissue and promotes adipocyte differentiation and lipid storage [36-38]. In mammals, PPARγ activates the expression of genes characteristic of mature adipocytes and adipogenesis, including FAS [37,38] and hence the expression of PPARγ, up-regulated in salmon fed VO, might be related to increased expression of FAS. However, increased PPARγ expression was only significant in Fat fish whereas FAS was significantly up-regulated only in Lean salmon. As fish PPARγ is functionally the most different of the three isotypes compared to mammalian PPARs, and is expressed more widely in fish tissues that in mammals, other mechanisms and functions may underlie the observed regulation [35].

In this study, the hypotriglyceridemic effect of LC-PUFA, well established in mammals [39,40], was also observed in salmon intestine. Lipogenesis was down-regulated in FO-fed fish, as demonstrated by decreased FAS expression (RT-qPCR) and the presence of a transcript containing a beta-ketoacyl synthase domain, a component of FAS (microarray). The differences in FAS expression were not as marked as in liver [9] and were only significant in Lean fish but, together with the LC-PUFA biosynthesis data, demonstrate the active role of salmon intestine in lipid metabolism [21]. However, despite up-regulation of lipogenesis by dietary VO, lipid accumulation in enterocytes was lower than in fish fed FO, contrary to previous reports of VO promoting lipid accumulation in enterocytes [16-18].

In contrast, the hypotriglyceridemic effect of LC-PUFA did not involve the typical increase in β-oxidation [39], reported in mice intestine [36,40]. As in liver, no changes were observed in the expression of β-oxidation genes carnitine palmitoyltransferase I (CPT1) and acyl-CoA oxidase (ACO) [10]. Nonetheless, effects of dietary lipid on energy metabolism were observed in intestine. In particular, UCP and transcripts involved in the mitochondrial electron transport chain, including components of cytochrome c oxidase, NADH1 and ubiquinol-cytochrome c reductase complexes, and the mitochondrial metabolite transporter SCaMC-2, were slightly down-regulated by dietary VO, possibly suggesting reduced energetic metabolism. EPA may act as a mitochondrial proliferator in both rat and salmon liver [41,42], which might also explain this result.

### Vegetable oils as potential sources of contaminants in alternative diets for aquaculture?

The use of FO in feeds has also been questioned in relation to levels of persistent organic pollutants, POPs [43], whereas VO are generally considered safer alternatives to dietary FO [44]. However, results suggested that VO had greater impact on xenobiotic metabolism in salmon intestine than FO, with the transcriptome and proteome both showing up-regulation of transcripts and proteins involved in detoxification and protection from oxidative stress in fish fed VO. This was surprising, considering expression of these genes has been associated with FO supplementation and higher levels of organic contaminants and/or increased peroxidative susceptibility of LC-PUFA [22,45,46]. In particular, and linked to a detoxification role, up-regulation of CYP1A transcript and epoxide hydrolase 2 (EPHX2) protein was found in fish fed VO. Cytochrome P450 1A metabolizes many exogenous and endogenous molecules, including pollutants (e.g., polychlorinated biphenyls-PCBs, polycyclic aromatic hydrocarbons-PAHs, dioxins and dibenzofurans) as well as several metabolic products (e.g., steroids, bile acids). Its expression is sensitive to low levels of contaminants and therefore it is a commonly used marker [47,48]. Lipid peroxidation and antioxidant enzymes are also biomarkers for environmental xenobiotic contamination in fish, as the catalytic actions of detoxifying enzymes like CYP1A produce high levels of reactive oxygen species (ROS) [49,50]. We observed up-regulation of CAT and of a selenoprotein transcript, as well as of HPX and PRDX1 proteins in salmon fed VO. CAT and PRDX1 catalyze the decomposition of hydrogen peroxide [51], hemopexin prevents heme-mediated oxidative stress [52], and selenoproteins have diverse roles, including selenium transport and antioxidant defense [53]. Expression of other genes involved in protection from oxidative stress like GST and SOD were not affected, but MT-A was down-regulated in the Lean family group fed VO. MT-A, besides having an antioxidant role, also protects against heavy metal toxicity and maintains physiological zinc homeostasis [54] and hence could be responding to contaminants more abundant in FO (e.g., heavy metals) [55].

Previous analysis of contaminants in comparable feeds using the same standard FO and a similar VO blend, showed that levels of several POPs, including organochlorine pesticides, and heavy metals were substantially lower, but polycyclic aromatic hydrocarbons (PAH) were 10-fold increased in the VO diet compared to the FO diet [55]. PAH derive from incomplete combustion of organic compounds and can be found in high concentrations in fats, including VO, through multiple routes of contamination, before, during or after oil processing [56]. They are metabolized by both CYP1A1 and EPHX2, among other enzymes, and CYP1A1 is induced by PAH in mammals [57]. Therefore, higher PAH levels in the VO diet might explain, at least partly, the results obtained in the present study although, unlike POPs, PAHs are not persistent and are readily eliminated from fish tissues [58].

High doses of PAH result in significant intestinal hyperplasia in fish, with an increase in cell proliferation and faster epithelial turnover [59]. Previous studies on intestinal gene expression in fish indicated a reduction in cell proliferation or differentiation associated with dietary FO replacement by VO [21,22], possibly due to lower levels of membrane LC-PUFA and reduced oxidative stress [22]. In the present study, no major impact on cell proliferation was apparent in the intestinal transcriptome or proteome data. Two transcripts related to cell proliferation, PA2G4 and cyclin G1, were slightly down-regulated in fish fed VO, but in mammals these have opposing effects [60,61] and, furthermore, two mammalian PA2G4 isoforms have been shown to have opposite effects in cellular proliferation [62] and hence results are inconclusive.

Previously, expression of caspases, effectors of controlled cell death or apoptosis, was affected by replacement of dietary FO by VO in fish [21,22]. Apoptosis is particularly important in organs with high rates of cellular turnover such as intestine but, in addition to maintaining normal gut function, apoptosis may be affected by pathological or toxic conditions, including those induced by environmental chemical contaminants [63,64]. In the present study, expression of CASP3B was up-regulated in salmon fed VO, particularly in the Lean family group and a similar, non-significant trend was observed for CASP6A/B. As ROS are important signalling molecules in apoptotic processes, these results could be linked to a cytotoxic effect causing increased oxidative stress in VO [64]. Relevant to the above was the up-regulation of galectin 2 (LGALS2) in the proteome of salmon fed VO. Galectins are pleiotropic regulators of immune functions and are up-regulated by injury and infectious conditions, have well-recognized modulatory roles in mammalian intestinal inflammatory diseases, and their mode of action involves induction of apoptosis [65,66]. The lack of major effects on cell proliferation and only slight up-regulation of CASP3 and LGALS2 suggests that any contaminant doses experienced by the fish were unlikely to have caused any serious morphophysiological damage in the intestine. As similar trends were not seen in the hepatic transcriptome of these individuals [9], this may suggest intestine can potentially metabolize and detoxify xenobiotics present in the diet. Furthermore, there were no growth or general performance issues with these fish [5]. Therefore, the data do not imply abnormal gastrointestinal functions or effects on final product quality.

### Effect of genotype in intestinal transcriptome and proteome

Contrary to diet, genotype did not have a major impact on metabolism genes, apart from transcripts related to the proteasomal degradation pathway including a strong down-regulation of PSMB8 in Lean fish, particularly fed VO. This gene has been recently found to have a molecular evolution history that suggests a very strong selective pressure for its functional dimorphism to be maintained in vertebrates [67]. Two different alleles, A-type and F-type, can be found in basal vertebrate species, including Atlantic salmon. The PSMB8F lineage was lost in common ancestors of higher teleosts and tetrapods but was then independently revived *de novo* through the appearance of F-type alleles within the PSMB8A lineage. In this study we did not find evidence of significant differences between families groups for the A-type allele in the transcriptomic analysis as the probe showing significant variation between families in the microarray corresponded to the PSMB8F allele. Hence, it was also the F-type transcript that was validated by RT-qPCR, using type-F specific primers. However, further studies would be required to confirm this and to assess the functional significance of this result. On the other hand, expression of PSMB1 was down-regulated in the intestine proteome of Lean fish. Proteolysis through this pathway is essential for many cellular processes, including the cell cycle, signalling, cellular defence and responses to oxidative stress [68]. Therefore, this response might be related to defence against cellular stress, as another difference between the two family groups was related to xenobiotic and oxidant metabolism. Apart from lower expression of a CYP1A transcript in Lean fish, two proteins with antioxidant roles, HPX and PRDX, were down-regulated in the proteome of Lean compared to Fat fish. Alpha globin, or haemoglobin alpha (HBA), a major component of blood and potent mediator of oxidative stress, can have both protective and damaging effects depending on complex interactions in H_2_O_2_-rich environments [69]. However, given its opposite regulation to HPX, whose main role is to scavenge heme and protect from its toxic effects [52], up-regulation of HBA in Lean fish may indicate heme-mediated oxidative stress.

The apoptotic pathway may be differentially affected by genotype, with down-regulation of CASP3, VDAC2 and ANXA4 in the Lean family group, the latter two transport proteins having well-recognized roles in apoptosis [70,71]. In contrast, heat shock proteins that protect against environmental stresses were increased in the intestine transcriptome and proteome of Lean salmon. This response could be associated with the observed changes in the ubiquitin-proteasome degradation system, as the systems have been functionally coupled in mammals. Thus, moderate exposure to a heat shock can cause a transient increase in intracellular proteolysis by the ubiquitin-proteasome pathway, followed by a phase of slower or even inhibited protein degradation [72,73]. Furthermore, Pirkkala et al. [74] demonstrated transcriptional induction of heat shock genes when proteasome activity was down-regulated. However, judging by the fold-changes, these effects are only relevant when fish were fed VO, and hence could be more related to dietary changes. Collectively, the data may indicate higher sensitivity of Lean fish to environmental or endogenous stresses due to replacement of dietary FO by VO.

The predominant influence of genotype was in the expression of intestinal transcripts of structural proteins, particularly collagen alpha chains, but also osteonectin, TAGLN, troponin I and keratocan, which were up-regulated in Lean fish, whereas troponin C was down-regulated. Furthermore, CNN1 and TAGLN were down-regulated in the intestinal proteome in Lean fish. Collagen, the main component of connective tissue, helps to maintain the structural integrity of tissues [75], while osteonectin is an extracellular matrix glycoprotein with high affinity towards collagen and whose expression has been associated with remodelling processes in tissues, including human intestine during development/morphogenesis and in diseased mucosa [76]. Troponin, TAGLN and CNN1 are all involved in actin binding, actin-myosin interaction and muscle contraction. The inverse regulation of troponins is not conflicting as they have different roles in actomyosin cross-bridge formation and contraction; binding of troponin C to Ca^2+^ induces conformational changes that counteract the inhibitory action of troponin I [77]. Expression of TAGLN transcript and protein showed opposite effects but a lack of correlation between transcriptomic and proteomic data is not unprecedented [78]. As discussed above, this result might also be explained by the presence of similar duplicated genes in Atlantic salmon that are regulated differently. Transcriptomic results were validated by RT-qPCR for COL1A2, although only significantly when fish were fed the VO diet, for which fold changes were higher. In addition, in the microarray results differences in expression of structural proteins between family groups were consistently more accentuated in fish fed VO which could suggest a cumulative effect of diet. Furthermore, MYO was up-regulated in fish fed VO compared to FO but only in Lean fish (RT-qPCR), and significant diet × genotype interactions were found for alpha-actinin 1, tubulin beta-2 chain and procollagen-lysine 2-oxoglutarate 5-dioxygenase 2 (Additional file 1), which were up-regulated in Lean salmon, compared to Fat, but only when fed VO. In cod, replacement of FO by VO resulted in changes in intestinal expression of structural genes with the potential to alter the structural and mechanical properties of the intestinal muscle layer, including a range of actin-binding transcripts [21].

The present study is the first investigation of the influence of genetic background of families with different flesh adiposity phenotypes on intestinal gene expression of a fish species. Effects were subtle and consequently their potential impacts difficult to fully assess. However, if genetic selection for families better adapted to alternative formulations is an approach taken in the future, the potential for genotype-specific differences being exacerbated when VO replaces dietary FO should be further examined to assess the consequences of these changes in intestinal gene expression.

## Conclusions

Metabolic activity, particularly lipid and energy, of intestinal tissue responded to dietary lipid composition but was also affected by genotype. The LC-PUFA biosynthesis pathway, typically up-regulated when salmon are fed VO, was especially influenced by genetic background. The Lean fish showed an enhanced response to low dietary n-3 LC-PUFA and the expression of Δ5fad, Δ6fad, elovl5b and elovl2 in the intestine showed high plasticity and was reflected in tissue biochemical composition indicating that their transcriptional regulation might be under feedback control by n-3 LC-PUFA, mainly DHA. Lower n-3 LC-PUFA in VO increased lipogenesis in Lean salmon, assessed by expression of FAS, while β-oxidation appeared unaffected, although transcripts involved in mitochondrial respiratory or electron transport chains were down-regulated, suggesting reduced activity in fish fed VO. Higher expression of genes and proteins involved in xenobiotic metabolism (CYP1A and EPHX2), antioxidant defence (CAT, HPX and PRDX1), and apoptosis (Casp3B) were observed in VO-fed fish, suggesting they might be responding to higher levels of contaminants, particularly PAH, in the diet. However, the intestine appeared able to metabolize and detoxify xenobiotic substances potentially present in the diet without major deleterious effects. Nonetheless, the data suggest that further attention should be given to contaminants in VO in the future. On the other hand, the data indicate potential genotype-specific differences in the response of the intestinal transcriptome and proteome to dietary VO. These include potential changes in structural properties of the intestinal layer and defence against cellular stress suggesting the Lean group was more susceptible to diet-induced oxidative stress. Considering the possibility of selecting families better adapted to alternative diet formulations, and the central role of intestine as a major barrier to nutrients, contaminants and pathogens, greater attention should be given to this organ when evaluating the effects of diet and genotype.

## Methods

### Feeding trial and sampling

A dietary trial was conducted using two genetically characterized groups of Atlantic salmon (*Salmo salar*) post-smolts comprising full-sib families selected from a breeding program (Landcatch Natural Selection Ltd., Argyll, Scotland). The choice of the family groups was based on estimated breeding values (EBVs) of the parents for high or low flesh adiposity, assessed by Torry Fatmeter (Distell Industries, West Lothian, UK), a trait that was found to have a heritability ranging from 0.17 to 0.39 in this dataset. The two groups were created from four unrelated full-sib families; two families from the extreme lower end of the EBV distribution for flesh lipid content (Lean) and two families from the extreme upper end of the distribution (Fat). The average EBV for the lipid content of the Fat families was 2.00 percentage units higher than that of the Lean families, representing a standardised selection differential of 2.33 standard deviations. Assessment of the flesh and visceral lipid contents at the end of the trial confirmed differences in adiposity between the groups [5].

Two thousand fish of each group were stocked into eight 12 x 5m^3^ net pens at the Ardnish Fish Trials Unit (Marine Harvest Scotland, Lochailort, Scotland; 500 fish pen^-1^). Duplicate pens of each group were fed one of two experimental diets (Skretting ARC, Stavanger, Norway) containing 25-32% fish meal, 40-45% plant meals and 27.5-30% oil (percentages varying according to pellet size) supplied either as standard northern FO or as a VO blend comprising rapeseed, palm and *Camelina* oils in a ratio of 5:3:2. Diets were formulated to fully satisfy the nutritional requirements of salmonid fish [79] and contained similar levels of PUFA but different n-3 and n-6 PUFA contents, 25.3% and 4.6% in the FO diet and 13.4% and 17.1% in the VO diet, respectively [5]. After 55 weeks, 25 fish per pen were sampled 24 h after the last meal. Fish were killed by a blow to the head following anaesthesia, and intestinal tissue (pyloric caeca) collected, immediately frozen in liquid nitrogen and stored at −70°C prior to analyses. Further details can be found in Bell et al. [5].

### Lipid extraction and fatty acid analyses

Total lipid from 1 g of intestine of four fish per treatment was extracted and determined gravimetrically [80], and fatty acid methyl esters (FAME) prepared by acid-catalysed transesterification of total lipid [81]. FAME were separated and quantified by gas chromatography as described in detail previously [5]. Significant differences in intestinal fatty acid composition were determined by two-way ANOVA (p<0.05) using the SPSS 16.0 statistical package (SPSS Inc., Chicago IL, USA).

### RNA extraction and purification

Intestinal tissue (0.2 g) from six individuals per experimental group was homogenised in 2mL TRI Reagent and total RNA isolated following manufacturer’s instructions (Ambion, Applied Biosystems, Warrington, U.K.). RNA quantity and quality (integrity and purity) were assessed by gel electrophoresis and spectrophotometry (NanoDrop ND-1000, Thermo Scientific, Wilmington, U.S.A.), and 100 μg of total RNA from each sample further cleaned by mini spin-column purification (RNeasy Mini Kit, Qiagen, Crawley, UK).

### Microarray hybridizations, image processing and statistical analysis

The TRAITS/SGP (v.2.1) salmon 17k cDNA microarray described by Taggart et al. [33] was used (ArrayExpress accession: A-MEXP-1930). A dual-labelled experimental design was employed, with each sample being competitively hybridised against a common pooled-reference. The experiment comprised 2 genotypes (Lean/Fat) × 2 diets (VO/FO) × 6 biological replicates. Indirect labelling was employed for preparing the microarray targets. Antisense amplified RNA (aRNA) was produced from 500 ng of purified total RNA per sample using the Amino Allyl MessageAmpTM II aRNA Amplification Kit as per manufacturer’s instructions (Ambion, Applied Biosystems), followed by Cy3 (for samples) or Cy5 (for pooled reference) fluor (GE HealthCare, Buckinghamshire, U.K.) incorporation through dye-coupling reaction [10]. Microarray hybridizations were performed in a Lucidea semi-automated system (GE Healthcare) without pre-hybridization. For each array, every labelled biological replicate and corresponding pooled reference (40 pmol each dye, c. 150 ng aRNA) were combined and added to the hybridization solution. Two post-hybridization automatic washes followed by six manual washes to a final stringency of 0.1× SSC (EasyDipTM Slide staining system; Canemco Inc., Quebec, Canada) were performed before scanning.

Scanning was performed at 10 μm resolution using an Axon GenePix 4200AL Scanner (MDS Analytical Technologies, Wokingham, Berkshire, U.K.). Laser power was constant (80%) and “auto PMT” was enabled to adjust each channel at less than 0.1% feature saturation and Cy3/Cy5 mean intensity close to one. BlueFuse software (BlueGnome, Cambridge, U.K.) was used to identify features and extract fluorescence intensity values from TIF images. The resulting fluorescence intensity data (BlueFuse proprietary algorithm) and quality annotations for the 17,102 gene features were exported into the GeneSpring GX version 10.0.2 analysis platform (Agilent Technologies, Wokingham, Berkshire, U.K.) after undergoing block Lowess normalization. All control features were excluded from subsequent analyses. Data transformation and quality filtering were as in Morais et al. [9,10]. This gave a final list of 15,498 genes that were eligible for statistical analysis. Experimental annotations complied fully with minimum information about a microarray experiment (MIAME) guidelines [82] and experimental hybridisations are archived on the EBI ArrayExpress database (http://www.ebi.ac.uk/arrayexpress/) under accession number E-TABM-1173. Hybridization data were analysed in GeneSpring by two-way ANOVA, which examined the explanatory power of the variable diet and genotype and the interaction between the two, followed by Gene Ontology (GO) enrichment analysis of the significant lists of features, at a significance level of 0.05. No multiple test correction was employed, as previous analyses, confirmed by RT-qPCR, indicated that such corrections are over-conservative for this type of data [9,32].

### RT-qPCR gene expression analysis

Expression of selected genes, for microarray validation and to further examine biological processes of interest, was studied by reverse transcription quantitative real time PCR (RT-qPCR), with target qPCR primer sequences given in Additional file 2. In addition, amplification of two reference genes, *cofilin-2* and elongation factor-1α (*elf-1α*), was performed. One μg of column-purified total RNA per sample was reverse transcribed into cDNA using the VersoTM cDNA kit (ABgene, Surrey, U.K.) using a mixture of random hexamers (400ng/μL) and anchored oligo-dT (500ng/μL) at 3:1 (v/v). Negative controls (no enzyme) were performed to check for genomic DNA contamination. A similar amount of cDNA was pooled from all samples and the remaining cDNA diluted 20-fold with water. RT-qPCR analysis used relative quantification with the amplification efficiency of each primer pair assessed by serial dilutions of the cDNA pool. Amplifications were carried out in duplicate using a Quantica machine (Techne, Cambridge, U.K.) in a final volume of 20 μl containing 2–8 μl diluted cDNA, 0.5 μM of each primer and 10 μl AbsoluteTM QPCR SYBR® Green mix (ABgene), with a systematic negative control (NTC-non template control). The qPCR profiles contained an initial activation step at 95°C for 15 min, followed by 30–40 cycles: 15 s at 95°C, 15 s at the specific primer pair annealing temperature (Ta; Additional file 2) and 15 s at 72°C. After amplification, a melt curve was performed confirming a single product in each reaction, RT-qPCR product sizes checked by agarose gel electrophoresis, and identity of amplicons confirmed by sequencing. Gene expression was analysed using the relative expression software tool (REST 2008, http://www.gene-quantification.info/), employing a pair wise fixed reallocation randomisation test (10,000 randomisations) with efficiency correction [83].

### Protein extraction and labelling

Six intestine samples (100 mg) per treatment were rapidly disrupted by homogenization and sonication on ice in 1 ml of DIGE lysis/labeling buffer (7M Urea, 2M Thiourea, 4% CHAPS and 30mM Tris, pH 8.5) in the presence of 10 μl of a protease inhibitor cocktail (ref. P8340, Sigma-Aldrich) and 4μl of 250 mM EDTA. After centrifugation at 12,000 × g for 20 min at 4°C, the supernatant was recovered and protein concentration determined (Quick Start Bradford Protein Assay Kit, ref. # 500–0202, Bio-Rad). Protein (500 μg) was purified by precipitation (ReadyPrepTM 2-D Cleanup kit, Bio-Rad) and the pellet re-suspended in DIGE lysis/labeling buffer at ~5μg/μl.

Samples were labelled using CyDye DIGE fluors (5nmol labelling kit; GE Healthcare), following manufacturer’s instructions. Three of the experimental replicates of each treatment (50 μg protein with pH corrected to 8.0-8.5) were labelled individually with 400 pmol Cy3 and the remaining three with 400 pmol Cy5. In addition, equal amounts of all experimental samples were pooled and 600 μg of protein (50 μg per 2-D gel) were batch-labelled with Cy2. The three labelled samples, corresponding to two experimental samples and one internal reference pool, were then combined to have in each 2-D gel samples corresponding to fish fed either FO or VO within the same family group.

### Two-dimensional (2-D) polyacrylamide gel electrophoresis

Rehydration buffer (ReadyPrep 2-D starter kit, Bio-Rad) containing 0.2% DTT was added to the pooled protein samples to a final volume of 450 μl, which were loaded onto Immobiline DryStrip pH 3–11 NL, 24 cm IPG strips (GE Healthcare) by passive rehydration at room temperature overnight in the dark. Proteins were separated in the first dimension by isoelectric focusing at 20°C (Ettan IPGphor, GE Healthcare), applying increasing voltage until 200 V for 4 h, increasing to 500 V over a period of 3 h, then keeping the applied tension at a constant 1000 V for 1 h, followed by a further increase to 8000 V over 90 min, maintaining this voltage for almost 9 h (total of 79,200 Vh). After isoelectric focusing the strips were equilibrated in two 40 min steps using 50mM Tris–HCl pH 8.8, 6M urea, 30% glycerol, 2% SDS buffer, to which 2 % DTT (w/v) and 2.8% iodoacetamide (GE Healthcare) were added to produce reducing and alkylating buffers, respectively. The strips were loaded onto a 12.5% acrylamide gel cast between low fluorescence glass cassettes (EttanDALTsix gel caster system, GE Healthcare). The strips were overlaid with ReadyPrep Overlay Agarose (Bio-Rad) and the six gel cassettes run in the EttanDALT system in two steps: at 60 mA, 80 V, 6 W (upper limits) for 1 h, and then 240 mA, 500 V, 78 W until the bromophenol blue dye front had run to ~1 cm above the bottom of the gels. Laemmli buffers (250mM Tris, 1.92M glycine, 1% SDS) were used in the lower (1X) and upper (2X) chambers, respectively.

### Gel imaging and analysis

Labelled gels were scanned using a Typhoon TRIO (GE Healthcare) and Cy2, Cy3 and Cy5 images acquired using 520BP40, 580BP30 and 670BP30 laser emission filters, respectively, at 500 PMT and 100 μm resolution. Images were cropped to remove extraneous areas prior to analysis (ImageQuantTL, GE Healthcare), and image analysis performed using DeCyder V7.0 (GE Healthcare). The estimated number of spots for each co-detection procedure was set at 10,000 and an exclusion filter was applied to remove spots with a volume lower than 30,000. Differential expression of protein spots was examined by two-way ANOVA at a significance level of 0.05. After verifying that significant spots were well matched across the gels, two pick lists were generated with a total of 22 and 45 spots for the diet and genotype factors, respectively (Figure 1; Additional files 3 and 4).

### Spot picking and protein identification by peptide fragment fingerprinting

Four preparative gels were run under the conditions described above but with higher amounts of protein (600 and 1000 μg). They were stained with colloidal Coomassie and, whenever possible, spots were excised and sequenced in the Mass Spectrometry Laboratory ITQB-UNL (Oeiras, Portugal), where in-gel digestion and extraction of the proteins from the gel was performed, followed by micropurification, and peptides identified by mass spectrometry (using an Applied Biosystems (ABI) 4800 MALDI TOF/TOF Analyzer). The search engine MASCOT was then used to identify and confirm protein ID’s from the peptide mass fingerprinting (PMF; MS) and peptide fragment fingerprinting (PFF; MS/MS) data.

## Competing interests

The authors declare that they have no competing interests.

## Authors’ contributions

SM performed laboratory work and data analysis, with assistance from TS and OC in proteomics; DRG was responsible for family selection; JBT and JEB supported the microarray analysis; PR supported the proteomic analysis; SM wrote the first draft of the manuscript, followed by contributions from remaining authors; SM, JGB and DRT planned and coordinated the research; JGB and DRT were project leaders. All authors read and approved the final manuscript.

## Supplementary Material

Additional file 1 Intestine transcripts corresponding to the top 100 most significant features exhibiting a significant diet × family interaction. Click here for file

Additional file 2 **Primers used for RT-qPCR analyses [**[84-89]**].** Click here for file

Additional file 3 2D-gels pick list for the factor diet. Click here for file

Additional file 4 2D-gels pick list for the factor genotype. Click here for file
