# Supplementary material for: Effects of genotype and dietary fish oil replacement with vegetable oil on the intestinal transcriptome and proteome of Atlantic salmon (Salmo salar)
Source: BMC Genomics. 2012 Sep 4;13:448. doi: 10.1186/1471-2164-13-448 (PMC3460786; doi:10.1186/1471-2164-13-448)
Supplement: Additional file 1 — Intestine transcripts corresponding to the top 100 most significant features exhibiting a significant diet × family interaction. [file 1471-2164-13-448-S1.doc]

**Additional file 1:** **Intestine transcripts corresponding to the top 100 most significant features exhibiting a significant diet × family interaction.** This table shows the annotated features (62% of all clones) arranged by categories of biological function and, within these, by decreasing significance (assessed by two-way ANOVA). Also indicated are the GenBank accession numbers for each clone (or, when not available, the probe number is given instead) and the expression ratios between fish fed VO and those fed FO, for each one of the families, and between Lean and Fat fish fed either FO or VO.

| **Accession no.** | **Gene** | **VO/FO** | |  | **Lean/Fat** | |  | **p-value** |
| --- | --- | --- | --- | --- | --- | --- | --- | --- |
|  |  | **Fat** | **Lean** |  | **FO** | **VO** |  |  |
| ***Metabolism (41%)*** | |  |  |  |  |  |  |  |
| *Lipid metabolism (12%)* | |  |  |  |  |  |  |  |
| DW589103 | ATP citrate lyase | 1.1 | - 1.3 |  | 1.3 | - 1.1 |  | 0.0002 |
| AM397481 | Delta-6 fatty acyl desaturase | 1.3 | - 1.1 |  | 1.2 | - 1.2 |  | 0.0004 |
| AY458652 | Delta-6 fatty acyl desaturase | 1.9 | - 1.4 |  | 1.6 | - 1.7 |  | 0.0005 |
| DW588445 | Delta-6 fatty acyl desaturase | 1.9 | - 1.3 |  | 1.5 | - 1.6 |  | 0.0005 |
| CK877956 | Triacylglycerol lipase | 1.4 | - 1.2 |  | 1.4 | - 1.2 |  | 0.0012 |
| AJ425157 | Phospholipase B-like 1 | - 1.3 | 1.6 |  | - 1.5 | 1.4 |  | 0.0013 |
| CK897463 | ATP citrate lyase | 1.2 | - 1.3 |  | 1.3 | - 1.2 |  | 0.0015 |
| AY458652 | Delta-6 fatty acyl desaturase | 2.2 | - 1.4 |  | 1.6 | - 1.9 |  | 0.0017 |
| CK888091 | Delta-6 fatty acyl desaturase | 1.8 | - 1.3 |  | 1.5 | - 1.6 |  | 0.0027 |
| CK885553 | Peroxisomal proliferator-activated receptor gamma | - 1.0 | 1.4 |  | - 1.3 | 1.1 |  | 0.0049 |
| *Energy metabolism/generation of precursor metabolites (12%)* | |  |  |  |  |  |  |  |
| CK896189 | Calcium-binding mitochondrial carrier protein SCaMC-2 | 1.1 | - 1.7 |  | 1.1 | - 1.6 |  | 0.0006 |
| CK890974 | Calcium-binding mitochondrial carrier protein SCaMC-2 | 1.0 | - 1.5 |  | - 1.1 | - 1.6 |  | 0.0010 |
| EG647452 | Cytochrome c oxidase subunit 4 isoform 1 | - 1.2 | 1.1 |  | - 1.1 | 1.1 |  | 0.0012 |
| CK886135 | Creatine kinase mitochondrial 1 | 1.1 | - 1.2 |  | 1.3 | - 1.1 |  | 0.0013 |
| GE624328 | Pyruvate dehydrogenase kinase, isoenzyme 2 | 1.5 | - 1.3 |  | 1.1 | - 1.6 |  | 0.0035 |
| *Protein and amino acid metabolism (10%)* | |  |  |  |  |  |  |  |
| AM042268 | N-acylaminoacyl-peptide hydrolase | - 1.1 | 1.3 |  | - 1.2 | 1.1 |  | 0.0011 |
| EG648672 | Aspartate aminotransferase | 1.2 | - 1.2 |  | 1.2 | - 1.2 |  | 0.0012 |
| DW590246 | Betaine aldehyde dehydrogenase | - 1.0 | - 1.4 |  | 1.2 | - 1.2 |  | 0.0012 |
| DW588095 | Heat shock protein DnaJ (Hsp40) homolog | - 1.1 | 1.2 |  | - 1.3 | 1.1 |  | 0.0029 |
| *Carbohydrate metabolism (5%)* | |  |  |  |  |  |  |  |
| EG649263 | Endosulfine alpha | - 1.0 | - 1.3 |  | 1.1 | - 1.2 |  | 0.0021 |
| CK885545 | Glucose transporter type 8 | 1.5 | 1.0 |  | 1.3 | - 1.1 |  | 0.0023 |
| *Xenobiotic and oxidant metabolism (2%)* | |  |  |  |  |  |  |  |
| CK884553 | UDP glucuronosyltransferase 2-like | 1.1 | - 1.3 |  | 1.1 | - 1.3 |  | 0.0011 |
| CK885392 | Catalase | 1.1 | 1.6 |  | - 1.4 | 1.0 |  | 0.0020 |
| ***Transport/ intracellular trafficking (7%)*** | |  |  |  |  |  |  |  |
| CK878806 | AP1 gamma subunit-binding protein 1 | - 1.2 | 1.2 |  | - 1.1 | 1.3 |  | 0.0001 |
| AJ425217 | Rhesus-associated glycoprotein | - 1.1 | 1.3 |  | - 1.2 | 1.2 |  | 0.0006 |
| AM041759 | Mitochondrial import inner membrane translocase subunit TIM13 | - 1.2 | 1.3 |  | - 1.3 | 1.2 |  | 0.0045 |
| ***Regulation of transcription (12%)*** | |  |  |  |  |  |  |  |
| DW588482 | General transcription factor 3C polypeptide 4 | 1.2 | - 1.2 |  | 1.1 | - 1.3 |  | 0.0004 |
| CN181253 | COMM domain-containing protein 7 | - 1.2 | 1.3 |  | - 1.3 | 1.3 |  | 0.0013 |
| CK879147 | High-mobility group box 1 | - 1.1 | 1.3 |  | - 1.1 | 1.3 |  | 0.0028 |
| CK889611 | DNA-directed RNA polymerase | - 1.2 | 1.2 |  | - 1.1 | 1.3 |  | 0.0039 |
| BM413765 | Inhibitor of DNA binding/differentiation 2B | - 1.0 | 1.5 |  | - 1.3 | 1.2 |  | 0.0041 |
| ***Translation (5%)*** | |  |  |  |  |  |  |  |
| CN181210 | 40S ribosomal protein S27 | 1.1 | - 1.2 |  | 1.3 | - 1.0 |  | 0.0005 |
| CK887633 | Ribosome biogenesis protein NSA2 | - 1.2 | 1.1 |  | - 1.3 | - 1.0 |  | 0.0036 |
| ***Signalling/Signal transduction (18%)*** | |  |  |  |  |  |  |  |
| CO471793 | Rho GDP-dissociation inhibitor 1 | 1.1 | - 1.1 |  | 1.3 | 1.0 |  | 0.0007 |
| CK885243 | Integrin-linked protein kinase | 1.3 | - 1.3 |  | 1.3 | - 1.3 |  | 0.0022 |
| CK885881 | Tetraspanin-9 | - 1.0 | 1.2 |  | - 1.0 | 1.1 |  | 0.0024 |
| EG647377 | Regulator of G-protein signaling 5 | - 1.2 | 1.1 |  | - 1.0 | 1.3 |  | 0.0024 |
| EG647545 | Protein tyrosine phosphatase receptor type D | - 1.1 | 1.1 |  | 1.0 | 1.2 |  | 0.0026 |
| CK890952 | G protein gamma subunit | 1.2 | - 1.1 |  | 1.1 | - 1.2 |  | 0.0041 |
| pit_cpi_C2F04 | Phosducin-like protein 3 | - 1.2 | 1.1 |  | - 1.3 | 1.1 |  | 0.0042 |
| CK885079 | Sonic hedgehog-like protein | 1.0 | 1.3 |  | - 1.3 | - 1.0 |  | 0.0048 |
| ***Structural proteins (15%)*** | |  |  |  |  |  |  |  |
| CO472006 | Cytokeratin-13 | - 1.3 | 1.0 |  | - 1.2 | 1.1 |  | 0.0002 |
| CK885977 | Cytokeratin-18 | 1.1 | - 1.1 |  | 1.1 | - 1.2 |  | 0.0004 |
| CN181168 | Type II keratin | 1.1 | - 1.1 |  | 1.1 | - 1.0 |  | 0.0016 |
| EG648864 | Alpha-actinin 1 | - 1.1 | 1.3 |  | - 1.1 | 1.3 |  | 0.0021 |
| EG647396 | Tubulin beta-2 chain | - 1.1 | 1.4 |  | - 1.1 | 1.3 |  | 0.0030 |
| CK884318 | Procollagen-lysine 2-oxoglutarate 5-dioxygenase 2 | - 1.2 | 1.3 |  | - 1.2 | 1.3 |  | 0.0032 |
| CK876976 | Beaded filament structural protein 2 | 1.1 | - 1.1 |  | 1.1 | - 1.1 |  | 0.0045 |
| ***Immune response (2%)*** | |  |  |  |  |  |  |  |
| CK881225 | Nonspecific cytotoxic cell receptor protein-1 | 1.2 | - 1.3 |  | 1.2 | - 1.2 |  | 0.0012 |
| ***Miscellaneous*** | |  |  |  |  |  |  |  |
| CK876833 | Keratocan | - 1.2 | 1.4 |  | - 1.0 | 1.6 |  | 0.0003 |
| DW592240 | Death-associated protein 1 | - 1.2 | 1.0 |  | - 1.2 | 1.1 |  | 0.0010 |
| CK895964 | Transposase | 1.1 | - 1.1 |  | 1.2 | - 1.0 |  | 0.0013 |
| BE518538 | Transformer 2 alpha homolog | 1.3 | - 1.1 |  | 1.1 | - 1.2 |  | 0.0015 |
| CK882254 | ADAM8 precursor (A disintegrin and metalloproteinase domain 8) | 1.0 | - 1.1 |  | 1.1 | - 1.1 |  | 0.0015 |
| CK882779 | 40 kDa peptidyl-prolyl cis-trans isomerase | 1.2 | - 1.2 |  | 1.2 | - 1.2 |  | 0.0018 |
| kid_cki_A1E04 | S100-like calcium binding protein | 1.2 | - 1.1 |  | 1.1 | - 1.3 |  | 0.0023 |
| BI468158 | Nuclear protein 1 | 1.4 | - 1.1 |  | - 1.0 | - 1.6 |  | 0.0025 |
| DW588616 | Kainate receptor | 1.6 | - 1.4 |  | 1.4 | - 1.6 |  | 0.0025 |
| CK882262 | Endonuclease domain-containing 1 | 1.2 | - 1.3 |  | 1.4 | - 1.1 |  | 0.0040 |
| CK877382 | Septin 7 | - 1.1 | 1.1 |  | - 1.0 | 1.2 |  | 0.0045 |
| EG649429 | Mitofusin-2 | 1.2 | - 1.3 |  | 1.2 | - 1.3 |  | 0.0046 |
| CK891845 | Transmembrane and coiled-coil domain-containing protein1 | 1.1 | - 1.3 |  | 1.2 | - 1.2 |  | 0.0049 |
