# Supplementary material for: Effects of genotype and dietary fish oil replacement with vegetable oil on the intestinal transcriptome and proteome of Atlantic salmon (Salmo salar)
Source: BMC Genomics. 2012 Sep 4;13:448. doi: 10.1186/1471-2164-13-448 (PMC3460786; doi:10.1186/1471-2164-13-448)
Supplement: Additional file 2 — Primers used for RT-qPCR analyses [[84-89]]. [file 1471-2164-13-448-S2.doc]

**Additional file 2: Primers used for RT-qPCR analyses.**

| Transcript | Primer sequence | | | Fragment | | Ta | Efficiency | | Accession No. | | Source | |
| --- | --- | --- | --- | --- | --- | --- | --- | --- | --- | --- | --- | --- |
| Δ5fad | GTGAATGGGGATCCATAGCA | | | 192 bp | | 56ºC | 0.990 | | AF478472 ^1^ | | [84] | |
|  | AAACGAACGGACAACCAGA | | |  | |  |  | |  | |  | |
| NADH1 | CGCTTTCCTCACCTTACTCG | | | 162 bp | | 60ºC | 0.988 | | TC161581 ^2^ | | New design | |
|  | AATAGGAAGGGGGAGGAGGT | | |  | |  |  | |  | |  | |
| UCP2 | GTTCATATATCTCGTCTTCACCACATC | | | 73 bp | | 60ºC | 0.994 | | BQ035332 ^1^ | | [85] | |
|  | CTGCATTCGGTGCAGGATT | | |  | |  |  | |  | |  | |
| MTHFD1 | TTAGGCGCTTCTCTCCCATA | | | 216 bp | | 60ºC | 0.997 | | NM_001165368 ^1^ | | New design | |
|  | CTCGCACATGTCCTTTCTCA | | |  | |  |  | |  | |  | |
| PA2G4 | AGACCGAAGCTGCGAAATAA | | | 209 bp | | 60ºC | 0.998 | | TC120403 ^2^ | | New design | |
|  | TCACCCGGTACAGTCTGACA | | |  | |  |  | |  | |  | |
| RP60S | AGATCAGGCTGCCAATGTCT | | | 93 bp | | 60ºC | 1.000 | | TC154862 ^2^ | | New design | |
|  | CTCTCATCAAGGCTGCCAAT | | |  | |  |  | |  | |  | |
| MYO | GGCCAAGAAGGCTATCACTG | | | 248 bp | | 60ºC | 0.997 | | TC129320 ^2^ | | New design | |
|  | CCGCGTCTACACCTCTTCTC | | |  | |  |  | |  | |  | |
| PCNA | CGTGGAGAGTATGGATTCGTCCCACG | | | 80 bp | | 60ºC | 0.996 | | GO059618 ^1^ | | [22] | |
|  | CGCAGCGGTAAGAGTCGAAC | | |  | |  |  | |  | |  | |
| CYP1A | CCAACTTACCTCTGCTGGAAGC | | | 68 bp | | 62ºC | 0.999 | | AF361643 ^1^ | | [86] | |
|  | GGTGAACGGCAGGAAGGA | | |  | |  |  | |  | |  | |
| ABCA1 | GGACGAACCCTGTGTCTGTT | | | 203 bp | | 60ºC | 0.993 | | EG836783 ^1^ | | [10] | |
|  | ATTTGCATTGCGTTTCAGTG | | |  | |  |  | |  | |  | |
| PSMB8 | CGTTTGCGGGATATCAGATT | | | 204 bp | | 60ºC | 0.999 | | TC138982 ^2^ | | New design | |
|  | GTGCCATGCTCCAGGTTAAT | | |  | |  |  | |  | |  | |
| TAGLN | AGGCTGGCATGACTGGATAC | | | 192 bp | | 60ºC | 0.999 | | TC146580 ^2^ | | New design | |
|  | TTCTCCTTGGTCCAGTTTGG | | |  | |  |  | |  | |  | |
| COL1A2 | CATTGCACCTTTGGACATTG | | | 219 bp | | 60ºC | 0.975 | | TC148395 ^2^ | | New design | |
|  | TCAGTCCATTGGTCCTCTCC | | |  | |  |  | |  | |  | |
| Δ6fad_a | CCCCAGACGTTTGTGTCAG | | | 181 bp | | 56ºC | 0.998 | | AY458652 ^1^ | | [84] | |
|  | CCTGGATTGTTGCTTTGGAT | | |  | |  |  | |  | |  | |
| elovl5a | ACAAGACAGGAATCTCTTTCAGATTAA | | | 137 bp | | 60ºC | 0.983 | | AY170327 ^1^ | | [34] | |
|  | TCTGGGGTTACTGTGCTATAGTGTAC | | |  | |  |  | |  | |  | |
| elovl5b | ACAAAAAGCCATGTTTATCTGAAAGA | | | 141 bp | | 60ºC | 0.999 | | DW546112 ^1^ | | [34] | |
|  | CACAGCCCCAGAGACCCACTT | | |  | |  |  | |  | |  | |
| elovl2 | CGGGTACAAAATGTGCTGGT | | | 145 bp | | 60ºC | 0.997 | | TC91192 ^2^ | | [34] | |
|  | TCTGTTTGCCGATAGCCATT | | |  | |  |  | |  | |  | |
| FAS | GTGCCCACTGAATACCATCC | | | 212 bp | | 60ºC | 0.999 | | CK876943 ^1^ | | [9] | |
|  | ATGAACCATTAGGCGGACAG | | |  | |  |  | |  | |  | |
| ACO | AAAGCCTTCACCACATGGAC | | | 230 bp | | 60ºC | 0.986 | | TC49531 ^2^ | | [32] | |
|  | TAGGACACGATGCCACTCAG | | |  | |  |  | |  | |  | |
| CPT1 | CCTGTACCGTGGAGACCTGT | | | 212 bp | | 60ºC | 0.998 | | AM230810 ^1^ | | [32] | |
|  | CAGCACCTCTTTGAGGAAGG | | |  | |  |  | |  | |  | |
| PPARα | TCCTGGTGGCCTACGGATC | | | 111 bp | | 60ºC | 0.980 | | DQ294237 ^1^ | | [87] | |
|  | CGTTGAATTTCATGGCGAACT | | |  | |  |  | |  | |  | |
| PPARβ | GAGACGGTCAGGGAGCTCAC | | | 151 bp | | 60ºC | 0.999 | | AJ416953 ^1^ | | [87] | |
|  | CCAGCAACCCGTCCTTGTT | | |  | |  |  | |  | |  | |
| PPARγ | CATTGTCAGCCTGTCCAGAC | | | 144 bp | | 60ºC | 0.991 | | AJ416951 ^1^ | | [87] | |
|  | TTGCAGCCCTCACAGACATG | | |  | |  |  | |  | |  | |
| GST | ATTTTGGGACGGGCTGACA | | | 81 bp | | 60ºC | 0.996 | | GE619558 ^1^ | | [22] | |
|  | CCTGGTGCTCTGCTCCAGTT | | |  | |  |  | |  | |  | |
| MT-A | CCTTGTGAATGCTCCAAAACTG | | | 101 bp | | 60ºC | 0.993 | | GO049284 ^1^ | | [22] | |
|  | CAGTCGCAGCAACTTGCTTTC | | |  | |  |  | |  | |  | |
| CAT | GGGCAACTGGGACCTTACTG | | | 59 bp | | 60ºC | 0.993 | | GE781438 ^1^ | | [22] | |
|  | GCATGGCGTCCCTGATAAA | | |  | |  |  | |  | |  | |
| SOD | CCACGTCCATGCCTTTGG | | | 140 bp | | 60ºC | 0.922 | | BG936553 ^1^ | | [88] | |
|  | TCAGCTGCTGCAGTCACGTT | | |  | |  |  | |  | |  | |
| CASP3B | TGCGATCAAAGTGTTCTCGAGTTT | | | 85 bp | | 62ºC | 0.997 | | GE794765 ^1^ | | [89] | |
|  | GGAAAGCAGCTGTTGTATCTGTTG | | |  | |  |  | |  | |  | |
| CASP6A/B | AGTGCAGGCCTTTGATAATCTGAAG | | | 215 bp | | 62ºC | 0.994 | | DQ008068 ^1^ | | [89] | |
|  | ACACGAAGCAGTCTGCATCTG | | |  | |  |  | |  | |  | |
|  |  | | |  | |  |  | |  | |  | |
| Reference genes | |  |  | |  | | |  | |  | |  |
| Elf-1α | CTGCCCCTCCAGGACGTTTACAA | | | 175 bp | | 60ºC | 0.995 | | AF321836 ^1^ | | [34] | |
|  | CACCGGGCATAGCCGATTCC | | |  | |  |  | |  | |  | |
| Cofilin-2 | AGCCTATGACCAACCCACTG | | | 224 bp | | 60ºC | 1.000 | | TC63899 ^2^ | | [34] | |
|  | TGTTCACAGCTCGTTTACCG | | |  | |  |  | |  | |  | |

^1^ GenBank (<http://www.ncbi.nlm.nih.gov/>)

^2^ Atlantic salmon Gene Index (<http://compbio.dfci.harvard.edu/tgi/>)
