# Supplementary material for: Effects of genotype and dietary fish oil replacement with vegetable oil on the intestinal transcriptome and proteome of Atlantic salmon (Salmo salar)
Source: BMC Genomics. 2012 Sep 4;13:448. doi: 10.1186/1471-2164-13-448 (PMC3460786; doi:10.1186/1471-2164-13-448)
Supplement: Additional file 3 — 2D-gels pick list for the factor diet. [file 1471-2164-13-448-S3.doc]

**Additional file 3:** **2D-gel pick list for the factor diet.** Table indicates protein spots identified, by two-way ANOVA (P<0.05), as being differentially expressed in salmon fed VO and FO diets, independently of the genotype.

| Spot No. | Average ratio (VO/FO) | |  | Two-Way ANOVA P-value | | |
| --- | --- | --- | --- | --- | --- | --- |
|  | Lean | Fat |  | Diet factor | Family factor | Interaction |
| 1151 | 1.38 | 1.54 |  | 0.0001 | 0.0120 | 0.5500 |
| 1148 | 1.32 | 1.56 |  | 0.0002 | 0.0270 | 0.3200 |
| 1135 | 1.24 | 1.27 |  | 0.0012 | 0.0200 | 0.8700 |
| 2114 | -1.17 | -1.58 |  | 0.0022 | 0.2900 | 0.1300 |
| 2275 | 1.58 | 1.11 |  | 0.0100 | 0.0990 | 0.0580 |
| 2687 | -1.2 | -1.64 |  | 0.0130 | 0.8000 | 0.1700 |
| 2195 | 1.58 | 1.12 |  | 0.0150 | 0.1900 | 0.0680 |
| 3014 | -1.39 | -1.24 |  | 0.0160 | 0.1700 | 0.5600 |
| 1119 | -1.25 | -1.17 |  | 0.0170 | 0.1100 | 0.6400 |
| 3168 | -1.46 | -1.18 |  | 0.0170 | 0.5500 | 0.2900 |
| 3445 | 1.17 | 1.15 |  | 0.0170 | 0.9100 | 0.8300 |
| 1136 | 1.23 | 1.4 |  | 0.0190 | 0.0660 | 0.4100 |
| 3108 | -1.43 | -1.19 |  | 0.0210 | 0.6800 | 0.3400 |
| 3334 | -1.48 | -1.18 |  | 0.0210 | 0.9500 | 0.3000 |
| 2266 | 1.09 | 1.11 |  | 0.0230 | 0.0800 | 0.8000 |
| 2766 | -1.66 | -1.03 |  | 0.0240 | 0.0620 | 0.0390 |
| 3331 | -1.58 | -1.18 |  | 0.0250 | 0.8600 | 0.2300 |
| 2932 | -2.08 | -1.22 |  | 0.0270 | 0.0024 | 0.0920 |
| 2683 | 1.14 | 1.09 |  | 0.0310 | 0.9500 | 0.7300 |
| 3253 | -1.44 | -1.22 |  | 0.0330 | 0.7000 | 0.5800 |
| 3109 | -1.34 | -1.25 |  | 0.0450 | 0.4100 | 0.8700 |
| 3243 | -1.23 | -1.11 |  | 0.0490 | 0.1600 | 0.5000 |
