# Supplementary material for: Effects of genotype and dietary fish oil replacement with vegetable oil on the intestinal transcriptome and proteome of Atlantic salmon (Salmo salar)
Source: BMC Genomics. 2012 Sep 4;13:448. doi: 10.1186/1471-2164-13-448 (PMC3460786; doi:10.1186/1471-2164-13-448)
Supplement: Additional file 4 — 2D-gels pick list for the factor genotype. [file 1471-2164-13-448-S4.doc]

**Additional file 4:** **2D-gel pick list for the factor genotype.** Table indicates protein spots identified, by two-way ANOVA (P<0.05), as being differentially expressed in Fat and Lean salmon family groups, independently of diet.

| Spot No. | Average ratio (Fat/Lean) | |  | Two-Way ANOVA P-value | | |
| --- | --- | --- | --- | --- | --- | --- |
|  | FO | VO |  | Diet factor | Family factor | Interaction |
| 2970 | 1.51 | 1.44 |  | 0.1400 | 0.0000 | 0.7000 |
| 2488 | 1.51 | 1.29 |  | 0.4500 | 0.0002 | 0.2300 |
| 2718 | -1.24 | -1.27 |  | 0.2600 | 0.0004 | 0.8600 |
| 2109 | -1.55 | -1.29 |  | 0.5400 | 0.0008 | 0.3700 |
| 2522 | -1.26 | -1.16 |  | 0.8900 | 0.0009 | 0.4200 |
| 2941 | 1.68 | 1.82 |  | 0.3200 | 0.0011 | 0.7200 |
| 2499 | 1.37 | 1.27 |  | 0.7700 | 0.0021 | 0.7100 |
| 2932 | 1.55 | 2.66 |  | 0.0270 | 0.0024 | 0.0920 |
| 1389 | 1.28 | 1.33 |  | 0.5600 | 0.0026 | 0.7800 |
| 2998 | 1.62 | 3.34 |  | 0.0640 | 0.0040 | 0.0850 |
| 2487 | 1.28 | 1.31 |  | 0.6000 | 0.0050 | 0.7600 |
| 3341 | -1.03 | -1.31 |  | 0.2900 | 0.0052 | 0.0220 |
| 1641 | 1.22 | 1.26 |  | 0.7200 | 0.0065 | 0.9400 |
| 1441 | -1.28 | -1.20 |  | 0.4700 | 0.0066 | 0.6600 |
| 2903 | 1.62 | 1.99 |  | 0.1800 | 0.0069 | 0.4100 |
| 2343 | 1.35 | 1.22 |  | 0.4100 | 0.0071 | 0.6200 |
| 2527 | -1.12 | -1.11 |  | 0.9500 | 0.0074 | 0.9000 |
| 1461 | 1.46 | 1.28 |  | 0.7200 | 0.0090 | 0.7100 |
| 2258 | 1.2 | 1.09 |  | 0.9500 | 0.0120 | 0.3500 |
| 2215 | 1.14 | 1.31 |  | 0.5900 | 0.0130 | 0.3300 |
| 2717 | 1.29 | 1.12 |  | 0.3100 | 0.0140 | 0.3300 |
| 2179 | 1.32 | 1.15 |  | 0.8500 | 0.0140 | 0.3500 |
| 2882 | -1.07 | -1.34 |  | 0.3100 | 0.0160 | 0.1600 |
| 2533 | 1.26 | 1.06 |  | 0.2500 | 0.0160 | 0.1300 |
| 2282 | 1.24 | 1.19 |  | 0.9400 | 0.0160 | 0.9300 |
| 311 | -1.26 | -1.15 |  | 0.4700 | 0.0170 | 0.4400 |
| 1135 | 1.14 | 1.17 |  | 0.0012 | 0.0200 | 0.8700 |
| 2746 | 1.37 | 1.23 |  | 0.4200 | 0.0230 | 0.6700 |
| 2157 | 1.34 | 1.47 |  | 0.3900 | 0.0240 | 0.7300 |
| 878 | -1.59 | -1.22 |  | 0.0740 | 0.0250 | 0.4500 |
| 2455 | -1.11 | -1.44 |  | 0.7700 | 0.0260 | 0.3100 |
| 1714 | -1.16 | -1.77 |  | 0.1700 | 0.0260 | 0.2900 |
| 1148 | 1.09 | 1.29 |  | 0.0002 | 0.0270 | 0.3200 |
| 2116 | 1.33 | 1.18 |  | 0.0870 | 0.0280 | 0.4200 |
| 2458 | 1.14 | 1.11 |  | 0.8000 | 0.0300 | 0.8400 |
| 2213 | 1.15 | 1.24 |  | 0.7400 | 0.0300 | 0.5300 |
| 3457 | -1.03 | -2.09 |  | 0.4100 | 0.0320 | 0.0560 |
| 3345 | 1.32 | 1.10 |  | 0.8900 | 0.0330 | 0.3300 |
| 2677 | 1.61 | 1.35 |  | 0.9100 | 0.0350 | 0.5900 |
| 2154 | 1.34 | 1.51 |  | 0.5500 | 0.0350 | 0.8300 |
| 2494 | 1.18 | 1.12 |  | 0.7200 | 0.0370 | 0.5800 |
| 2743 | 1.25 | 1.29 |  | 0.2600 | 0.0460 | 0.8300 |
| 2345 | -1.29 | -1.20 |  | 0.6700 | 0.0460 | 0.5800 |
| 2138 | 1.13 | 1.22 |  | 0.8700 | 0.0460 | 0.5800 |
| 2134 | 1.19 | 1.09 |  | 0.7100 | 0.0460 | 0.5100 |
